# NbPIRIN promotes the protease activity of papain-like cysteine protease NbRD21 to inhibit Chinese wheat mosaic virus infection

**DOI:** 10.1371/journal.ppat.1013037

**Published:** 2025-04-02

**Authors:** Kaili Zhong, Gecheng Xu, Jingjing Shi, Peng Liu, Aizhu Tu, Mila Wu, Jiaqian Liu, Jianping Chen, Jian Yang

**Affiliations:** State Key Laboratory for Quality and Safety of Agro-Products, Key Laboratory of Biotechnology in Plant Protection of MARA, Zhejiang Key Laboratory of Green Plant Protection, Institute of Plant Virology, Ningbo University, Ningbo, China; University of Cologne: Universitat zu Koln, GERMANY

## Abstract

Papain-like cysteine proteases (PLCPs) play critical roles in regulating plant immunity against a range of pathogens and a series of cysteine protease inhibitors have been identified, however, relatively little research has been done on proteins that enhance the protease activity of PLCPs. Here, we identified a protein named NbPIRIN, the silencing of *NbPIRIN* promotes Chinese wheat mosaic virus (CWMV) infection, whereas the transgenic overexpression of *NbPIRIN* inhibits CWMV infection in *Nicotiana benthamiana*. Furthermore, we found that NbPIRIN interacts with papain-like cysteine protease (NbRD21) and increases its protease activity. We demonstrated that the silencing of *NbRD21* significantly increased host susceptibility to CWMV infection, whereas the transgenic overexpression of *NbRD21* increased host resistance. Interestingly, CWMV CRP was found to interact with both NbPIRIN and NbRD21, thus interfering with the interaction between NbPIRIN and NbRD21 and subsequently inhibiting the protease activity of NbRD21. Since wheat is the natural host of CWMV, we identified TaPIRIN and TaRD21 and found that they had functions similar to those of NbPIRIN and NbRD21 in the CWMV response. These results reveal a previously unreported offensive and defensive strategy between plants and viruses.

## Author summary

Papain-like cysteine proteases (PLCPs) play critical roles in regulating plant immunity. Here, we identified a protein named *NbPIRIN*, it inhibits Chinese wheat mosaic virus (CWMV) infection. Furthermore, we found that NbPIRIN interacts with papain-like cysteine protease NbRD21 and increases its protease activity. *NbRD21* could also inhibits CWMV infection. Interestingly, CWMV CRP interact with both NbPIRIN and NbRD21 to interfer with the interaction between NbPIRIN and NbRD21 and subsequently inhibiting the protease activity of NbRD21. In addition, we identified TaPIRIN and TaRD21 in wheat, and found that they had functions similar to those of NbPIRIN and NbRD21 in the CWMV response. These results reveal a previously unreported offensive and defensive strategy between plants and viruses.

## Introduction

PIRIN (a Cupin domain-containing PIRIN protein) proteins are highly conserved among mammals, plants, fungi and prokaryotic organisms and belong to the cupin domain-containing superfamily [1]. The PIRIN protein family encompasses proteins with diverse functions, but these functions remain largely elusive. In humans, pirin-NF-κB binding is responsible for the modulation of the DNA-binding properties of NF-κB. Pirin may act as a reversible functional switch for the nuclear factor κB (NF-κB) transcription factor to respond to changes in the redox levels of the cell nucleus [2]. In Arabidopsis, there are four PIRIN genes. AtPirin1 has a specific interaction with GPA1 1 (the sole Arabidopsis Ga-subunit) and acts as the Ga effector in the signaling mechanism that inhibits the ABA-mediated delay in germination [3]. AtPirin1 is also involved in the response to UV light, blue light and abscisic acid [4,5]. AtPirin2 can interact with the papain-like cysteine proteases (PLCPs) XCP2, RD21A, and RD21B and stabilize XCP2 through the inhibition of its autolysis to increase susceptibility to the vascular pathogen *Ralstonia solanacearum* in Arabidopsis. This study also showed that PIRIN2 inhibits XCP2 activity, but the inhibition is reversible and eventually results in stabilization of XCP2 [6]. AtPirin2 was found to be localized to cells adjacent to vessel elements and was characterized as a regulator of non-cell-autonomous lignification of xylem tissues. AtPirin2 was subsequently shown to inhibit the expression of both lignin biosynthetic genes and SCW-related transcription factors, suppressing S-type lignin accumulation in the neighborhood of xylem vessels [7]. The *PIRIN* gene family is found in other dicot species, including tomato (*Solanum lycopersicum*) and the parasitic plant *Triphysaria versicolor*. The transcription levels of LePirin dramatically increase during camptothecin-induced programmed cell death (PCD) [8]. TvPirin is a generalized transcription factor associated with the expression of a number of genes, some of which are involved in haustorium development. TvPirin is transcriptionally upregulated in roots after they are exposed to the haustorium-inducing substance 2,6-dimethoxybenzoquinone [9]. PIRIN homologs have also been found in the monocot species rice, barley and wheat. OsPIRIN was found to interact with salt-, ABA-, and drought-induced RING finger protein 1 (OsSADR1), which is an E3 ligase. The interaction between OsSADR1 and OsPIRIN mediates proteolytic activity via the 26S proteasome pathway, resulting in the degradation of OsPIRIN [10]. In barley, the transcription level of PIRIN is reportedly induced by pathogen-derived trichothecenes that can induce cell death in plants [11,12]. The PIRIN gene family in *Triticum aestivum* comprises 18 genes that are specifically expressed and play a role in stress responses [13].

Plants have evolved a sophisticated immune system to combat pathogen invasions [14]. Numerous studies have highlighted the critical roles that papain-like cysteine proteases (PLCPs) play in regulating plant immunity against a range of pathogens, including bacteria, fungi, oomycetes, nematodes, and insects [15–20]. Papain-like cysteine proteases (PLCPs), which share a conserved protease domain, are prominent enzymes in the plant apoplast that function critically in plant growth and development; these PLCPs are notable for being involved in protein maturation and degradation, plant senescence, seed germination, and programmed cell death (PCD) [21–23]. Moreover, PLCPs can function as central hubs in plant immunity, controlling key processes in the plant immune system by inducing systemic immunity and degrading pathogen-effector proteins [20,24]. Arabidopsis null mutants of RD21 are more susceptible to the fungal pathogen *Botrytis cinerea* [18]. Tomato RCR3 null mutants have lost resistance based on the Cf-2 resistance gene against the fungi *Cladosporium fulvum* and *Phytophthora infestans* and the nematode *Globodera rostochiensis* [25–27]. Arabidopsis RD19 null mutants are impaired in resistance to the bacterial pathogen *Ralstonia solanacearum* [28]. Wheat TaRD21A defends against WYMV by releasing a small peptide [29]. In turn, pathogens can secrete effectors that target and inhibit PLCP activities, and PLCPs are the common targets of pathogen effectors [17,30–32]. WYMV-encoded nuclear inclusion protease-a (NIa) suppresses TaRD21A activity to promote viral infection [29]. SCMV NIa-Pro interacts with CCP1 and inhibits its protease activity to allow efficient viral infection [33]. However, to date, our knowledge of the functions of PLCP in plant‒virus interactions and the mechanisms by which viruses counteract PLCP activities remain largely unexplored. The activity of proteolytic enzymes is commonly controlled by various kinds of protease inhibitors. Inhibitors of cysteine proteases are also produced by almost every group of living organisms, being responsible for the control of intracellular proteolytic activity [34]. A series of cysteine protease inhibitors have been identified but relatively little research has been done on cysteine protease enhancers. One result showed that the Arabidopsis PIRIN2 interacts with papain-like cysteine proteases XCP2 and RD21A, and increase their protease activities [6].

Chinese wheat mosaic virus (CWMV) is classified in the genus *Furovirus*, family *Virgaviridae*. It is transmitted by an obligate root-infecting fungus-like organism, *Polymyxa graminis* (order Plasmodiophorales), and causes yellow mosaic disease in wheat crops in China [35,36]. CWMV has a rigid rod-shaped virion that contains two single-stranded positive-sense RNA segments [36]. The larger RNA segment (known as RNA 1) encodes a 153 kDa protein (P153) with unknown functions, a 212 kDa RNA-dependent RNA polymerase domain (RdRp), and a 37 kDa cell-to-cell movement protein (MP). The smaller RNA segment (known as RNA 2) encodes a 19 kDa major Coat protein (CP), two minor CP-related proteins (i.e., a 23 kDa N-CP translated from an upstream CUG initiation codon and an 84 kDa CP-RT translated by an occasional read-through of the CP gene UGA termination codon), and a 19 kDa cysteine-rich protein (CRP) [36–39]. Full-length infection clones of CWMV RNAs have been constructed and used to study the functions of CWMV proteins [40]. CWMV CRP has been identified as a VSR protein that inhibits the spread of silencing signals [37]. According to recent studies, the CWMV CRP protein can be phosphorylated by SAPK7 during CWMV infection [41].

In the present study, we identified a PIRIN protein that interacts with papain-like cysteine protease NbRD21 and increases the protease activity of NbRD21 to inhibit CWMV infection. Silencing of PIRIN or RD21 significantly increased host susceptibility to CWMV infection, whereas overexpression of PIRIN or RD21 increased host resistance. Furthermore, CWMV CRP interacts with both PIRIN and RD21 to reduce the hydrolase activity of RD21 by interfering with the interaction between PIRIN and RD21.

## Results

### The expression level of *NbPIRIN* affects CWMV infection in *N. benthamiana*

**Fig 1:**
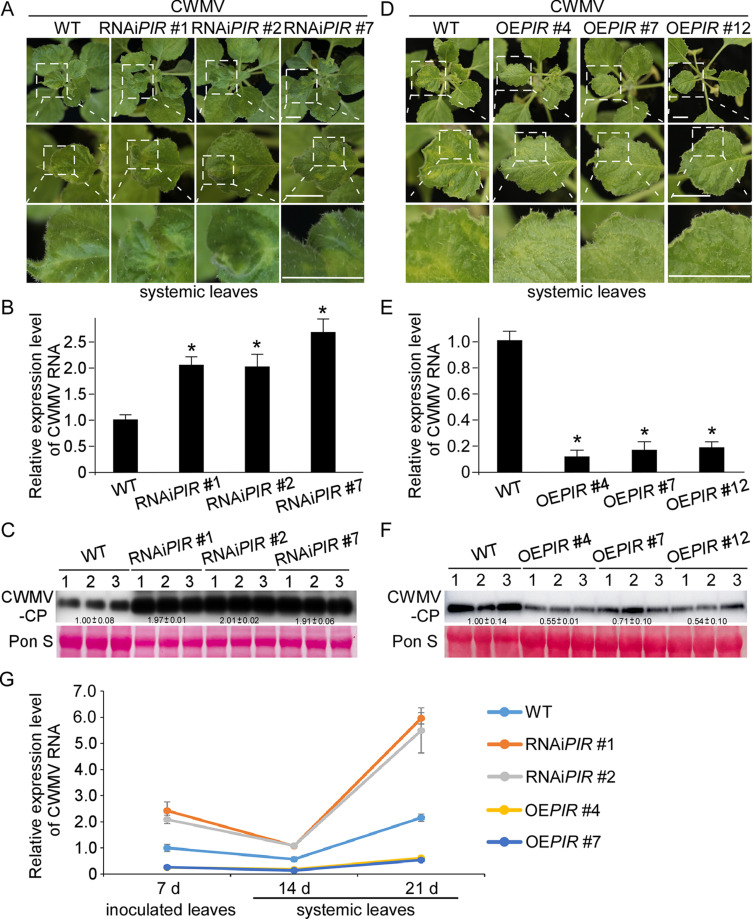
NbPIRIN is a positive regulator of plant immunity against CWMV infection. **(A)** Systemic mosaic symptoms in the CWMV-infected wild type and *NbPIRIN* RNAi-silenced lines. Photographs were taken at 21 days post CWMV inoculation (dpi). Scale bar = 2.5 cm. **(B)** Quantitative RT-PCR analysis of CWMV RNA accumulation in the CWMV-inoculated wild type, RNAi*PIR*#1, RNAi*PIR*#2 or RNAi*PIR*#7 plants. Asterisks above the bars indicate significant differences (*, **P** ≤ 0.05 by Student’s t-test). **(C)** Western blot analysis of CWMV CP accumulation using a CWMV CP specific antibody. The ponceau S-stained rubisco gel is used to show sample loadings. There were three biological replicates each consisting of a group of ten plants. **(D)** Images of leaves taken from wild type, *NbPIRIN* transgenic overexpression lines *OEPIR#*4, *OEPIR#*7 and *OEPIR#*12 plants at 21 dpi. Uninfected wild type *N. benthamiana* plants shown as control. Scale bar = 2.5 cm. **(E)** Quantitative RT-PCR analysis of CWMV RNA accumulation in the CWMV-inoculated wild type, *OEPIR*#4, *OEPIR*#7 and *OEPIR*#12 plants. Asterisks above the bars indicate significant differences (*, **P** ≤ 0.05 by Student’s t-test). **(F)** Western blot analysis of CWMV CP accumulation using a CWMV CP specific antibody. The ponceau S-stained rubisco gel is used to show sample loadings. There were three biological replicates each consisting of a group of ten plants. **(G)** Quantitative RT-PCR analysis of CWMV RNA accumulation in the CWMV-inoculated wild type, RNAi*PIR*#1, RNAi*PIR*#2, *OEPIR*#4 and *OEPIR*#7 plants at 7, 14 and 21 dpi. Inoculated leaves were collected at 7 dpi, systemic leaves were collected at 14 and 21 dpi. There were three biological replicates for each group.

PIRIN proteins have diverse functions, but their functions in the interaction between plants and viruses remain largely unknown. Here, we cloned the *NbPIRIN* gene (PQ728641.1) from *Nicotiana benthamiana*. Initially, we obtained three *NbPIRIN* RNAi-silenced lines in *N. benthamiana*, named RNAi*PIR*#1, RNAi*PIR*#2, and RNAi*PIR*#7, respectively. The results from reverse transcription quantitative polymerase chain reaction (RT-qPCR) indicated that the expression levels of *NbPIRIN* were significantly down-regulated in these RNAi-silenced lines (S1A Fig). Additionally, we observed that RNAi-mediated silencing of *NbPIRIN* adversely affected vegetative growth (S1B Fig). Given that the Chinese wheat mosaic virus (CWMV) can infect *N. benthamiana*, which is widely utilized as a model system for studying the interactions between viruses and plants, we investigated the role of NbPIRIN in CWMV infection in *N. benthamiana* by inoculating CWMV into wild-type (WT), RNAi*PIR*#1, RNAi*PIR*#2 and RNAi*PIR*#7 and plants through agroinfiltration. No mosaic symptoms were observed on the whole plants of WT, RNAi*PIR*#1, RNAi*PIR*#2 and RNAi*PIR*#7 at the age of inoculation (S1E Fig). After 21 days post inoculation (dpi), stronger mosaic symptoms were observed on the CWMV-inoculated RNAi*PIR*#1, RNAi*PIR*#2 and RNAi*PIR*#7 plants than on the CWMV-inoculated wild-type plants (Fig 1A). As a control, no mosaic symptoms were observed on WT, RNAi*PIR*#1, RNAi*PIR*#2 and RNAi*PIR*#7 plant leaves after 21 days post inoculation with agroinfiltration GV3101 (S1F Fig). Analyses of the accumulation of genomic RNA in the CWMV-inoculated plants via RT‒qPCR revealed that the RNAi*PIR*#1, RNAi*PIR*#2 and RNAi*PIR*#7 plants accumulated more CWMV genomic RNA than the wild-type plants did (Fig 1B). Analyses of CWMV CP (Coat protein) in the CWMV-inoculated plants via Western blotting yielded similar results (Fig 1C). To further investigate the effect of *NbPIRIN* on CWMV infection, we also obtained three *NbPIRIN* transgenic lines, named OE*PIR*#4, OE*PIR*#7, and OE*PIR*#12, respectively. The results of the RT-qPCR analysis indicated that the expression level of *NbPIRIN* was significantly upregulated in the *NbPIRIN* transgenic lines (S1B Fig). Additionally, we observed that the overexpression of *NbPIRIN* promoted vegetative growth (S1D Fig). No mosaic symptoms were observed in the whole plants of WT, OE*PIR*#4, OE*PIR*#7, and OE*PIR*#12 at the age of inoculation (S1G Fig). At 21 dpi, the plants inoculated with CWMV presented mosaic symptoms. However, the mosaic symptoms of the transgenic lines were weaker than those of the wild type (Fig 1D). As a control, no mosaic symptoms were observed on the plant leaves after 21 days post inoculation with agroinfiltration GV3101 (S1H Fig). The accumulation of CWMV genomic RNA revealed that OE*PIR*#4, OE*PIR*#7, and OE*PIR*#12 plants accumulated less CWMV genomic RNA than did the wild-type plants (Fig 1E). The results of the Western blot analysis were similar (Fig 1F). To further investigate the role of NbPIRIN in CWMV infection in *N. benthamiana*, we first analyzed the expression pattern of *NbPIRIN* in response to CWMV infection and found that *NbPIRIN* was upregulated after CWMV infection (S1I Fig). To investigate whether the observed resistance is merely a delay in infection or if it actually reduces viral load, we conducted a time-course experiment with sampling points at 7, 14, and 21 days post-CWMV inoculation. The RT-qPCR results confirmed the silencing or overexpression of *NbPIRIN* (S1J Fig). The findings indicated that CWMV successfully infected the inoculated leaves of wild type, *NbPIRIN* RNAi-silenced lines, and *NbPIRIN* transgenic lines at 7 dpi, and the viral load was reduced in the *NbPIRIN* transgenic lines, while it increased in the *NbPIRIN* RNAi-silenced lines at 7, 14, and 21 dpi (Fig 1G).

### NbPIRIN interacts with NbRD21, promoting its protease activity to inhibit CWMV infection

**Fig 2:**
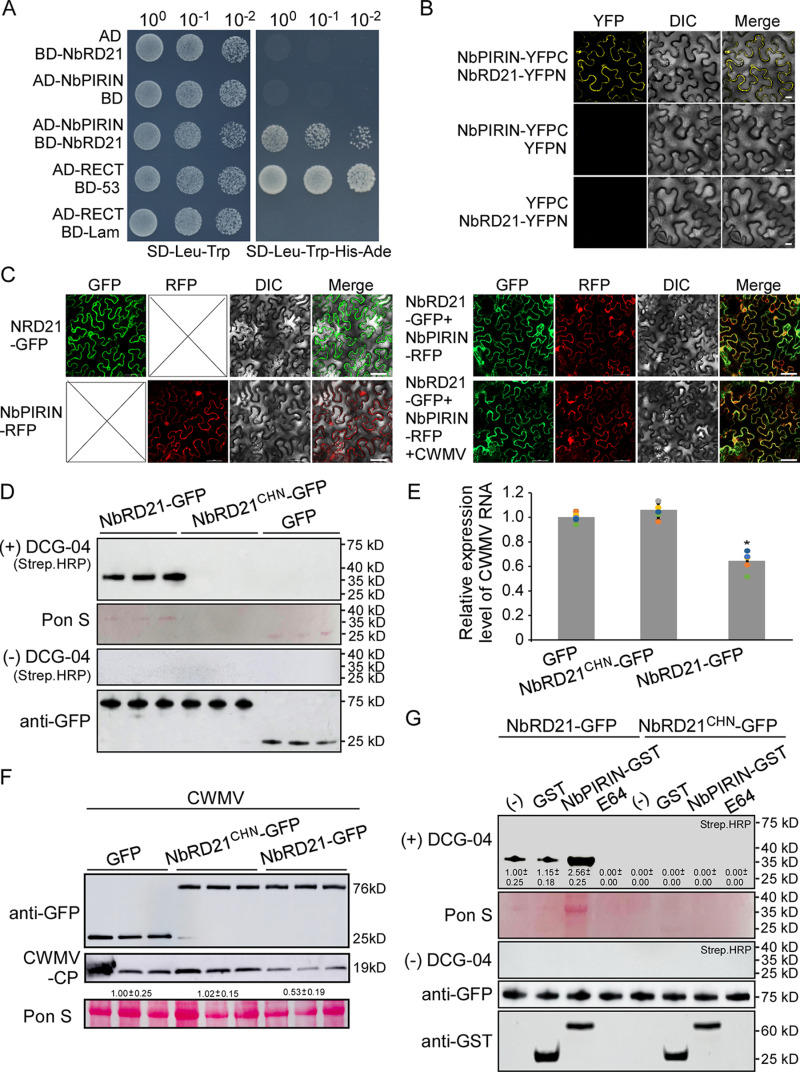
NbPIRIN interact with NbRD21 to promote its protease activity. **(A)** Yeast two hybrid assays for the interaction between NbPIRIN and NbRD21. The AD- and BD- plasmids were co-transformed into yeast AH109, and transformants were plated on SD-Leu-Trp for 3 days and transfer to selective SD-Leu-Trp-His-Ade for 5 days. AD-RECT and BD-53 were co-transformed as positive control, the AD-RECT and BD-lam were co-transformed as negative control. **(B)** BiFC assays confirming the interaction of NbPIRIN with NbRD21. NbPIRIN-YFPC was agro-injected together with NbRD21-YFPN into *N. benthamiana* leaves, and the samples were imaged by confocal microscopy at 48 hours post inoculation (hpi). Scale bar = 10 µm. NbPIRIN-YFPC agro-injected together with YFPN, and NbRD21-YFPN agro-injected together with YFPC were shown as negative controls. **(C)** Confocal fluorescence microscope (20× water, Nikon, Tokyo, Japan; A1+A1R) observation of NbPIRIN-RFP and NbRD21-GFP. Bar = 50 μm. **(D)** The NbRD21-GFP and NbRD21^CHN^-GFP were purified from apoplastic fluid that extracted from the leaves of *N. benthamiana* plants transiently expressing NbRD21-GFP or NbRD21CHN-GFP. GFP was purified from total protein that extracted from the leaves of *N. benthamiana* plants transiently expressing p35S-GFP. NbRD21-GFP, NbRD21^CHN^-GFP and GFP labeled with DCG-04 or not were detected with streptavidin-HRP. **(E)** Quantitative RT-PCR analysis of CWMV RNA accumulation in the *N. benthamiana* leaves transiently expressing NbRD21-GFP, NbRD21^CHN^-GFP or GFP. Asterisks above the bars indicate significant differences (*, **P** ≤ 0.05 by Student’s t-test). **(F)** Western blot analysis of NbRD21-GFP, NbRD21^CHN^-GFP or GFP in the *N. benthamiana* leaves using a GFP specific antibody. Western blot analysis of CWMV CP accumulation using a CWMV CP specific antibody. The ponceau S-stained rubisco gel is used to show sample loadings. The numbers below the bands mean the intensity ratio of the three biological replicates bands that calculated by Image J software. There were three biological replicates. **(G)** Effect of NbPIRIN on NbRD21 protease activity. The NbRD21-GFP and NbRD21^CHN^-GFP were purified from apoplastic fluid that extracted from the leaves of *N. benthamiana* plants transiently expressing NbRD21-GFP or NbRD21CHN-GFP. NbRD21-GFP or NbRD21^CHN^-GFP was labeled with DCG-04 or not in presence or absence of NbPIRIN-GST or E64, and detected with streptavidin-HRP. Proteins load were analyzed by Western blotting with anti-GFP antibodies (HT801, TransGen Biotech, China) and anti-GST antibodies (HT601, TransGen Biotech, China). The numbers below the bands mean the intensity ratio of the three repeated bands from three separate experiments that calculated by Image J software.

To investigate the biological function of NbPIRIN in CWMV infection, we screened an *N. benthamiana* cDNA library through yeast two hybrid (YTH) assays using BD-NbPIRIN as bait. After three independent screens, a total of 25 positive clones were obtained. After sequence analysis, these clones were found to encode 22 proteins (S2 Table). Three of these clones were found to encode a papain-like cysteine protease (KX375796.1). Since the result of phylogenetic analysis of *N. benthamiana* PLCPs with Arabidopsis PLCPs showed that papain-like cysteine protease 6 belongs to RD21 subfamily, we named it NbRD21 hereafter (S2A Fig). Since studies showed that PIRIN2 physically interacts with PLCPs (XCP2, RD21A, and RD21B) and stabilizes XCP2 to participate in full susceptibility to *R. solanacearum* in Arabidopsis [6]. We validate the interaction between NbPIRIN and NbRD21, a BD-NbRD21 plasmid was constructed and used in further yeast two-hybrid (YTH) assays. The results showed that AD-NbPIRIN interacts with BD-NbRD21 in yeast cells (Fig 2A). We further conducted a bimolecular fluorescence complementation (BiFC) assay and confirmed that NbPIRIN interacts with NbRD21 (Fig 2B). In addition, we analyzed the independent subcellular localization patterns of NbPIRIN and NbRD21, as well as their co-localization patterns under both CWMV-infected and uninfected conditions in *N. benthamiana* leaves. At 2 days post-infiltration, confocal microscopy revealed that NbPIRIN-RFP was present in both the nucleus and cytoplasm, while NbRD21-GFP was localized exclusively in the cytoplasm. Co-localization studies demonstrated that NbPIRIN and NbRD21 co-localized in the cytoplasm, and these co-localization patterns remained unchanged under CWMV infection conditions (Fig 2C). The PLCP protease domain contains a catalytic triad formed by the amino acids cysteine (Cys), histidine (His), and asparagine (Asn) [20]. To assess the protease activity of NbRD21 in the host, we mutated the three key hydrolase sites (C161A, H297A, and N317A) of NbRD21 to create the NbRD21CHN mutant, which served as a negative control. First, we conducted a protease activity profiling assay using the activity-based probe DCG-04, a biotinylated derivative of the irreversible PLCP inhibitor E-64 [42]. NbRD21-GFP, NbRD21CHN-GFP, or GFP was transiently overexpressed in *N. benthamiana* leaves, and the three proteins were purified using GFP-Trap magnetic agarose (gtma-20, Proteintech, USA) from leaf extracts. The three purified proteins were either supplemented with DCG-04 or left unsupplemented. After 60 minutes of incubation, a major band corresponding to the mature active protease was identified in the NbRD21-GFP samples on the blots using streptavidin-conjugated horseradish peroxidase (HRP). PLCPs are produced as pre-proproteases and contain an N-terminal signal peptide for secretion and an autoinhibitory prodomain that needs to be removed for protein activation, releasing a mature 25–35 kDa active protease [20]. The sizes of the proteins corresponded to the expected 35 kDa for the active protease [43]. The results indicated that NbRD21-GFP exhibits protease activity, whereas NbRD21^CHN^-GFP and GFP do not (Fig 2D). We then transiently overexpressed NbRD21-GFP, NbRD21^CHN^-GFP or GFP with CWMV in *N. benthamiana* leaves. At 7 dpi, the NbRD21-GFP overexpressing plants accumulated less CWMV genomic RNA than did the NbRD21^CHN^-GFP- or GFP-transiently overexpressing plants (Fig 2E). The results of the Western blot analysis revealed similar results (Figs 2F and S2B). To probe the nature of the interaction between NbPIRIN and NbRD21, we carried out protease activity profiling assays using purified NbRD21-GFP, with purified NbRD21^CHN^-GFP serving as a control. The samples were supplemented with or without DCG-04 and incubated with or without recombinant NbPIRIN-GST. The binding of DCG-04 was completely inhibited by prior treatment with excess E-64 before labeling. After 60 minutes of incubation, blots probed with streptavidin-conjugated horseradish peroxidase (HRP) showed one major band corresponding to the protease was detected in NbRD21-GFP incubated with or without GST, and a stronger band was identified when NbRD21-GFP incubated with NbPIRIN-GST. No major band corresponding to the protease was identified in NbRD21-GFP incubated with E-64 or in any samples of NbRD21^CHN^-GFP. These results indicate that NbPIRIN enhances the protease activity of NbRD21 (Figs 2G and S2C). To investigate whether NbPIRIN could stabilize NbRD21, we analyzed NbRD21 using an anti-GFP antibody at various time points following the addition of the protein translation inhibitor cycloheximide (CHX) and adenosine triphosphate (ATP) in the presence of either GST or NbPIRIN-GST. NbRD21-GFP was extracted from infiltrated *N. benthamiana* plants at 48 hours post-infiltration. The degradation rate of TaSRT2-GFP was comparable in the presence of GST or NbPIRIN-GST (S2D Fig). The results indicated that NbPIRIN does not stabilize NbRD21.

### NbRD21 positively regulates the resistance to CWMV infection in *N. benthamiana*

**Fig 3:**
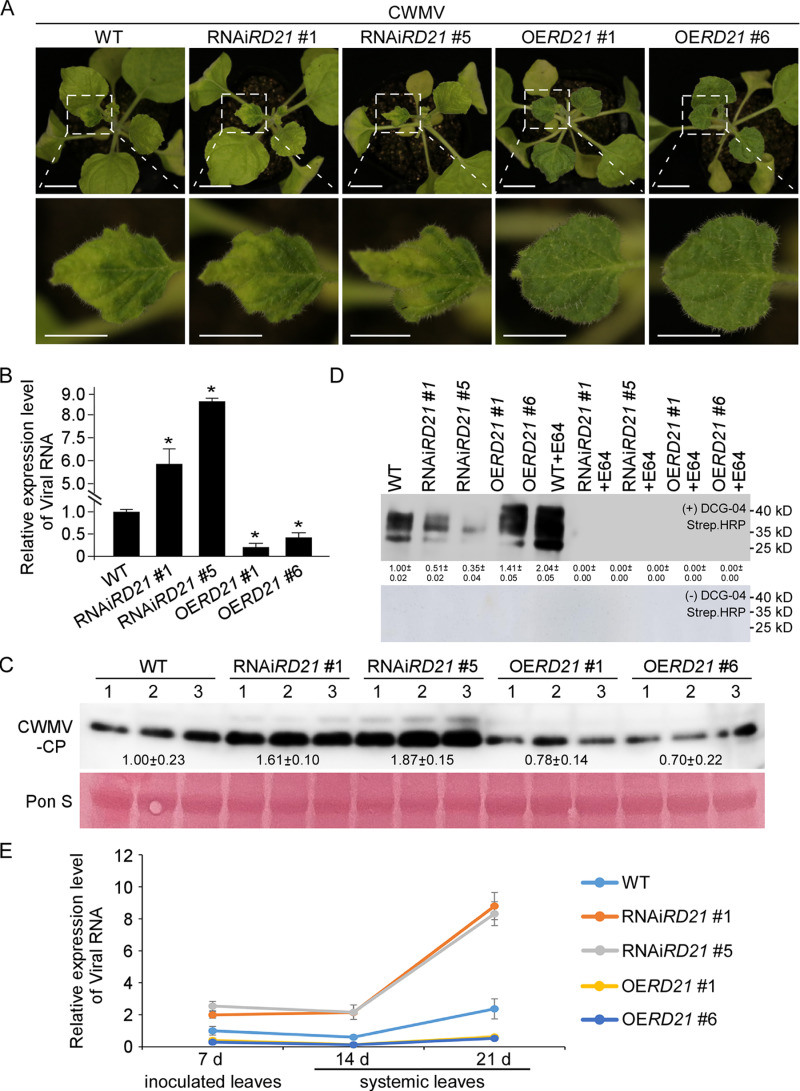
NbRD21 is a positive regulator of plant immunity against CWMV infection. **(A)** Systemic mosaic symptoms in the CWMV-infected wild type, RNA silencing *NbRD21* plants and *NbRD21* transgenic overexpression plants. Photographs were taken at 21 days post CWMV inoculation (dpi). Scale bar = 2 cm (upper panel), Scale bar = 1 cm (lower panel). **(B)** Quantitative RT-PCR analysis of CWMV RNA accumulation in the CWMV-inoculated wild type, RNAi*RD21*#1, RNAi*RD21*#5, *OERD21#1* or *OERD21#6* plants. Asterisks above the bars indicate significant differences (*, **P** ≤ 0.05 by Student’s t-test). **(C)** Western blot analysis of CWMV CP accumulation using a CWMV CP specific antibody. The ponceau S-stained rubisco gel is used to show sample loadings. There were three biological replicates each consisting of a group of ten plants. The intensity ratio was calculated by Image J software. **(D)** Apoplastic fluid from wild-type, RNAiRD21#1, RNAiRD21#5, OERD21#1 or OERD21#6 plants was labeled with DCG-04 in the presence or absence of E64. The numbers below the bands mean the intensity ratio of the bands that calculated by Image J software. **(E)** Quantitative RT-PCR analysis of CWMV RNA accumulation in the CWMV-inoculated wild type, RNAi*RD21*#1, RNAi*RD21*#5, OE*RD21*#1 and OE*RD21*#6 plants plants at 7, 14 and 21 dpi. Inoculated leaves were collected at 7 dpi, systemic leaves were collected at 14 and 21 dpi. There were three biological replicates for each group.

To investigate the role of NbRD21 in CWMV infection in *N. benthamiana*, we obtained two *NbRD21* RNAi-silenced lines (RNAi*RD21*#1 and RNAi*RD21*#5) and two NbRD21 transgenic lines (OE*RD21*#1 and OE*RD21*#6). The RT-qPCR results indicated that the expression level of *NbRD21* was significantly down-regulated in *NbRD21* RNAi-silenced lines and significantly up-regulated in *NbRD21* transgenic lines (S3A Fig). Additionally, we observed that RNAi-mediated silencing of *NbRD21* inhibited vegetative growth, whereas overexpression of *NbRD21* had no effect on vegetative growth (S3B Fig). No mosaic symptoms were observed on the whole plants of WT, RNAi*RD21*#1, RNAi*RD21*#5, OE*RD21*#1, and OE*RD21*#6 (S3C Fig). CWMV was inoculated into WT, RNAi*RD21*#1, RNAi*RD21*#5, OE*RD21*#1 and OE*RD21*#6 plants through agroinfiltration. At 21 dpi, stronger mosaic symptoms were observed on the CWMV-inoculated RNAi*RD21*#1 and RNAi*RD21*#5 plants than on the CWMV-inoculated wild-type plants. However, the mosaic symptoms of the transgenic lines were weaker than those of the wild type (Fig 3A). As a control, no mosaic symptoms were observed on WT, RNAi*RD21*#1, RNAi*RD21*#5, OE*RD21*#1 and OE*RD21*#6 plant leaves after 21 days post inoculation with agroinfiltration GV3101 (S3D Fig). Analyses of the accumulation of genomic RNA in the CWMV-inoculated plants via RT‒qPCR revealed that the RNAi*RD21*#1 and RNAi*RD21*#5 plants accumulated more CWMV genomic RNA than did the wild-type plants. However, OE*RD21*#1 and OE*RD21*#6 plants accumulated less CWMV genomic RNA than did the wild-type plants (Fig 3B). Analyses of CWMV CP in the CWMV-inoculated plants via Western blotting yielded similar results (Fig 3C). In addition, we performed a protease activity profiling assay of WT, RNAi*RD21*#1, RNAi*RD21*#5, OE*RD21*#1 and OE*RD21*#6, and the protein extract from these leaves was supplemented or not with DCG-04. After 60 min of incubation, one major band corresponding to the protease was identified. The results revealed that the protease activity was significantly increased in OE*RD21*#1 and OE*RD21*#6 plants, but significantly decreased in RNAi*RD21*#1 and RNAi*RD21*#5 plants, revealing that the protease activity of *NbRD21* is important for inhibitting CWMV infection (Fig 3D). To investigate whether resistance is merely a delay in infection or actually reduces viral load, we conducted a time-course experiment with sampling points at 7, 14, and 21 days post-CWMV inoculation. The RT-qPCR results confirmed the silencing or overexpression of *NbRD21* (S3E Fig). The findings indicated that CWMV successfully infected the inoculated leaves of wild-type, *NbRD21* RNAi-silenced lines, and *NbRD21* transgenic lines at 7 dpi. Notably, the viral load was reduced in *NbRD21* transgenic lines, while it increased in *NbRD21* RNAi-silenced lines at 7, 14, and 21 dpi (Fig 3E).

### Both NbPIRIN and NbRD21 interact with the CWMV CRP protein

**Fig 4:**
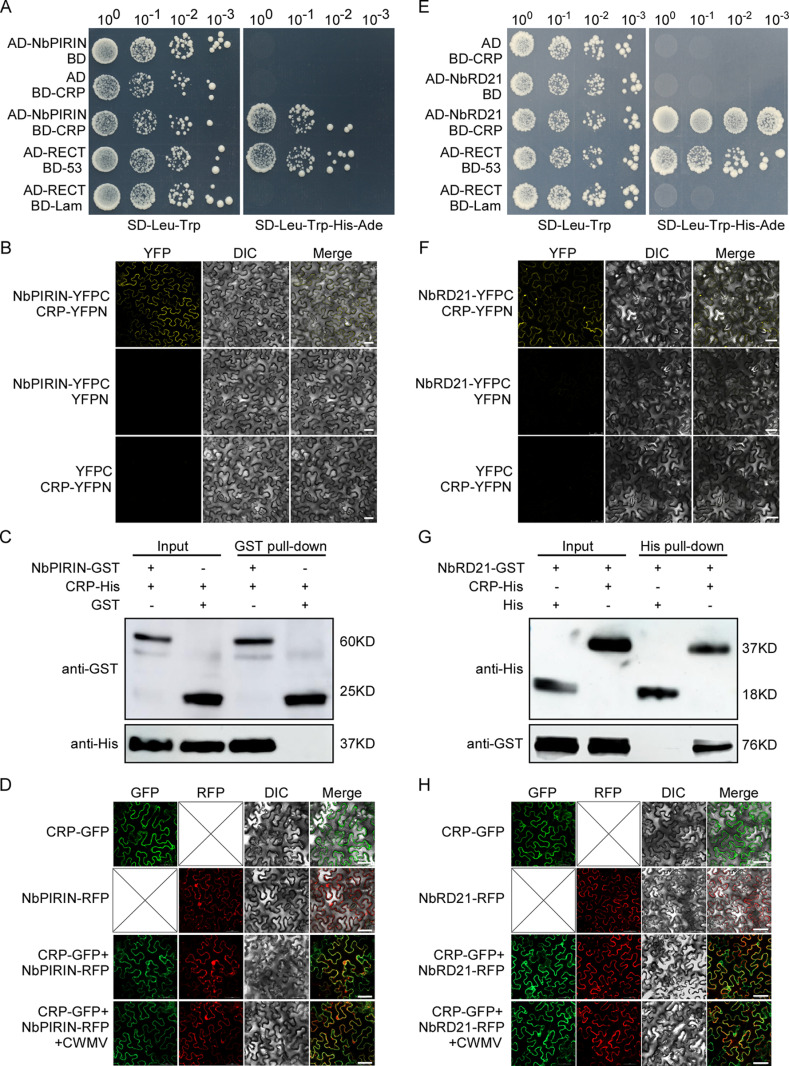
Both NbPIRIN and NbRD21 interact with CWMV CRP protein. **(A)** Yeast two hybrid assays for the interaction between NbPIRIN and CRP. The AD- and BD- plasmids were co-transformed into yeast AH109, and transformants were plated on SD-Leu-Trp for 3 days and transfer to selective SD-Leu-Trp-His-Ade for 5 days. AD-RECT and BD-53 were co-transformed as positive control, the AD-RECT and BD-lam were co-transformed as negative control. **(B)** BiFC assays confirming the interaction of NbPIRIN with CRP. NbPIRIN-YFPC was agro-injected together with CRP-YFPN into *N. benthamiana* leaves, and the samples were imaged by confocal microscopy at 48 hpi. Scale bar = 25 µm. NbPIRIN-YFPC agro-injected together with YFPN, and CRP-YFPN agro-injected together with YFPC were shown as negative controls. Each experiment was repeated three times with similar results. **(C)** GST pull down assays for NbPIRIN and CRP. NbPIRIN-GST and CRP-His were expressed and purified by affinity chromatography. Bound proteins were separated by SDS-PAGE in duplicate and analyzed by Western blotting with anti-His antibodies (HT501, TransGen Biotech, China) and anti-GST antibodies (HT601, TransGen Biotech, China). **(D)** Confocal fluorescence microscope (20× water, Nikon, Tokyo, Japan; A1+A1R) observation of NbPIRIN-RFP and CRP-GFP. Bar = 50 μm. **(E)** Yeast two hybrid assays for the interaction between NbRD21 and CRP. The AD- and BD- plasmids were co-transformed into yeast AH109, and transformants were plated on SD-Leu-Trp for 3 days and transfer to selective SD-Leu-Trp-His-Ade for 5 days. AD-RECT and BD-53 were co-transformed as positive control, the AD-RECT and BD-lam were co-transformed as negative control. **(F)** NbRD21-YFPC was agro-injected together with CRP-YFPN into *N. benthamiana* leaves, and the samples were imaged by confocal microscopy at 48 hpi. Scale bar = 25 µm. NbRD21-YFPC agro-injected together with YFPN, and CRP-YFPN agro-injected together with YFPC were shown as negative controls. Each experiment was repeated three times with similar results. **(G)** His pull-down assay was used to detect the interaction between NbRD21-GST and CRP-His. NbRD21-GST and CRP-His were expressed and purified by affinity chromatography. Bound proteins were separated by SDS-PAGE in duplicate and analyzed by Western blotting with anti-His antibodies (HT501, TransGen Biotech, China) and anti-GST antibodies (HT601, TransGen Biotech, China). **(H)** Confocal fluorescence microscope (20× water, Nikon, Tokyo, Japan; A1+A1R) observation of NbRD21-RFP and CRP-GFP. Bar = 50 μm.

To determine whether NbPIRIN directly interacts with CWMV-encoded protein(s) to affect infection, we performed yeast two-hybrid assays. The results revealed that CWMV cysteine-rich protein (CRP, sequence is shown in S1 Table), but not the other CWMV proteins, interacted with NbPIRIN (Fig 4A). To validate the interaction between NbPIRIN and CRP, we conducted bimolecular fluorescence complementation (BiFC) assays, which revealed that NbPIRIN and CRP interact in the cytoplasm (Fig 4B). Additionally, GST pull-down assays confirmed the interaction between NbPIRIN and CRP (Fig 4C). We also analyzed the independent subcellular localization patterns of NbPIRIN and CRP, as well as their co-localization patterns under both CWMV-infected and uninfected conditions in *N. benthamiana* leaves. At two days post-infiltration, confocal microscopy demonstrated that NbPIRIN-RFP was localized in both the nucleus and cytoplasm, while CRP-GFP was localized exclusively in the cytoplasm. Co-localization studies indicated that NbPIRIN and CRP co-localized in the cytoplasm, and these co-localization patterns remained unchanged under CWMV infection conditions (Fig 4D). Since NbPIRIN interacts with CRP and NbRD21 interacts with NbPIRIN, we speculated that CRP interacts with NbRD21. To confirm our suspicions, the AD-NbRD21 and BD-CRP plasmids were used in YTH assays. The results showed that AD-NbRD21 interacts with BD-CRP in yeast cells (Fig 4E). We further conducted BiFC assays between NbRD21 and CRP, and the results indicated that NbRD21 and CRP interact in the cytoplasm (Fig 4F). The HIS pull-down assays confirmed the interaction between NbRD21 and CRP (Fig 4G). Additionally, we analyzed the independent subcellular localization patterns of NbRD21 and CRP, as well as their co-localization patterns under both CWMV-infected and uninfected conditions in *N. benthamiana* leaves. At 2 days post-infiltration, confocal microscopy revealed that both NbRD21-RFP and CRP-GFP were present exclusively in the cytoplasm. Co-localization studies demonstrated that NbPIRIN and CRP co-localized in the cytoplasm, and these co-localization patterns remained unchanged under CWMV infection conditions (Fig 4H).

### CRP decreases the protease activity of NbRD21 by interfering with the interaction between NbPIRIN and NbRD21

**Fig 5:**
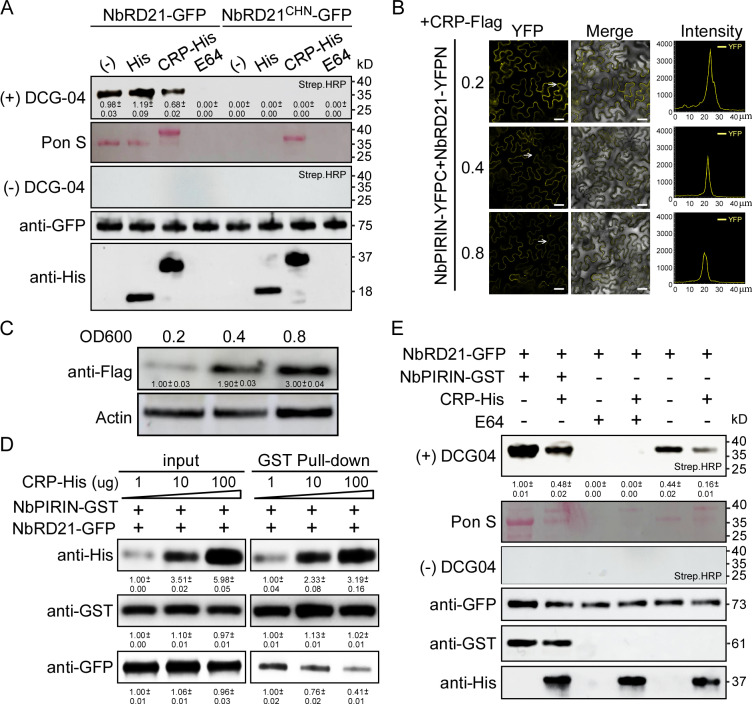
CRP interfering the interaction between NbPIRIN and NbRD21 to decrease the protease activity of NbRD21. **(A)** Effect of CRP on NbRD21 protease activity. The NbRD21-GFP and NbRD21^CHN^-GFP were purified from apoplastic fluid that extracted from the leaves of *N. benthamiana* plants transiently expressing NbRD21-GFP or NbRD21^CHN^-GFP. NbRD21-GFP or NbRD21^CHN^-GFP was labeled with DCG-04 or not in presence or absence of CRP-His or E64, and detected with streptavidin-HRP. The numbers below the bands mean the intensity ratio of the bands that calculated by Image J software. **(B)** NbPIRIN-YFPC was agro-injected together with NbRD21-YFPN and different concentrations of CRP-Flag into *N. benthamiana* leaves, and the samples were imaged by confocal microscopy at 48 hpi. Scale bar = 25 µm. The intensity indicates the strength of interaction. Each experiment was repeated three times with similar results. **(C)** Western blot analysis of CRP-Flag accumulation in the samples that mentioned in (C) using an anti-Flag specific antibody. The Actin is used to show sample loadings. The numbers below the bands mean the intensity ratio of the bands that calculated by Image J software. **(D)** Competitive binding experiment of CRP-His, NbPIRIN-GST and NbRD21-GFP. NbRD21-GFP was transiently infiltrated using agrobacterium in leaves of *N. benthamiana*, the CRP-His and NbPIRIN-GST proteins were purified from E. coli. Cultures of NbRD21-GFP and NbPIRIN-GST were pelleted to a same concentration, increasing amounts of CRP-His, and pulled down by GST beads. Immunoblots were performed using anti-GST, anti-GFP and anti-His antibodies to detect the associated proteins. Each experiment was repeated three times with similar results. The numbers below the bands mean the intensity ratio of the bands that calculated by Image J software. **(E)** The NbRD21-GFP was purified from apoplastic fluid that extracted from the leaves of *N. benthamiana* plants transiently expressing NbRD21-GFP. NbRD21-GFP was labeled with DCG-04 or not in presence or absence of CRP-His, NbPIRIN-GST or E64, and detected with streptavidin-HRP. Proteins load were analyzed by Western blotting with anti-GFP antibodies (HT801, TransGen Biotech, China), anti-His antibodies (HT501, TransGen Biotech, China) and anti-GST antibodies (HT601, TransGen Biotech, China). The numbers below the bands mean the intensity ratio of the bands that calculated by Image J software.

To probe the nature of the interaction between CRP and NbRD21, we also carried out protease activity profiling assays in *N. benthamiana* leaves transiently overexpressing NbRD21-GFP and purified using GFP-Trap magnetic agarose (gtma-20, Proteintech, USA) in conjunction with the activity-based probe DCG-04. Leaves transiently overexpressing NbRD21^CHN^-GFP served as controls. The purified protein samples were then combined with DCG-04 and incubated with or without recombinant CRP. E-64 and His were added as controls. After 60 min of incubation with DCG-04, one major band corresponding to the protease was identified on the blots using streptavidin-conjugated horseradish peroxidase (HRP). The results revealed that CRP significantly reduced DCG-04 labeling in NbRD21-GFP sample, indicating that CRP decreases the protease activity of NbRD21 (Fig 5A). In addition, we investigated whether NbRD21 is capable of cleaving CRP. CRP-GFP, RFP, and NbRD21-RFP were transiently expressed in *N. benthamiana* leaves and purified using GFP-Trap magnetic agarose (gtma-20, Proteintech, USA) or RFP-Trap magnetic agarose (rtma-20, Proteintech, USA), respectively. The amount and molecular weight of CRP-GFP did not change after incubation with RD21-RFP for 60 minutes compared to samples incubated with RFP (S4 Fig). These results indicate that NbRD21 does not cleave CRP. Since CRP interacts with both NbPIRIN and NbRD21, we suspected that CRP affects the interaction of NbPIRIN with NbRD21. To confirm our suspicions, we conducted a BiFC assay. NbRD21-YFPN, NbPIRIN-YFPC and CRP-Flag were transiently coexpressed in *N. benthamiana* leaves. As the concentration of *Agrobacterium* culture carrying CRP-Flag was increased from OD_600_ = 0.2 to 0.8, the intensity of YFP fluorescence in plant cells was reduced in a dose-dependent manner (Fig 5B and 5C). In addition, we conducted an in vitro pulldown assay of NbRD21-GFP and NbPIRIN-GST in the presence of different concentrations of CRP-His. Interestingly, with increasing concentrations of CRP added to the system, the enrichment of NbRD21-GFP in the GST-bound resins gradually decreased, suggesting that CRP disrupted the interaction between NbPIRIN and NbRD21 (Fig 5D). We further conducted a protease activity profiling assay to probe the effects of CRP and NbPIRIN on NbRD21 protease activity. NbRD21-GFP was transiently overexpressed in *N. benthamiana* leaves and purified use GFP-Trap magnetic agarose. NbRD21-GFP were combined with DCG-04 and incubated with or without recombinant CRP or NbPIRIN. The binding of DCG-04 was fully inhibited by treatment with excess E-64 prior to labeling. After 60 min of incubation with DCG-04, one major band corresponding to the protease was identified on the blots using streptavidin-conjugated horseradish peroxidase (HRP). The samples incubated with NbPIRIN presented the strongest band, and the samples incubated with CRP presented the weakest band. The samples incubated with both NbPIRIN and CRP presented a moderate-strength band similar to that of the NbRD21-GFP samples incubated without CRP or NbPIRIN (Fig 5E). These findings demonstrate that CRP affects the interaction between NbPIRIN and NbRD21, thus decreasing the protease activity of NbRD21.

### Wheat TaPIRIN promotes the protease activity of TaRD21 to inhibit CWMV infection

Since wheat is the natural host of CWMV, we searched the International Wheat Genome Sequencing Consortium database (http://plants.ensembl.org/index.html) and identified 15 copies of the *TaPIRIN*-like gene. To explore the phylogenetic relationships between TaPIRINs and NbPIRIN, sequences were used to construct a maximum likelihood phylogenetic tree via MEGA 7 (S5A Fig). Three TaPIRIN genes located on chromosomes 1A (TraesCS1A02G391900), 1B (TraesCS1B02G420000) and 1D (TraesCS1D02G40000) were identified. Amino acid sequence alignment suggested that these three genes had 45.45% similarity to NbPIRIN and that the PIRIN domain had 72.16% similarity (S5B Fig). Structural homology searches conducted between NbPIRIN and three TaPIRIN proteins revealed that the three TaPIRIN proteins exhibit a similar protein structure to NbPIRIN (S5C Fig). For convenience, TraesCS1A02G391900 was designated as TaPIRIN in this study, as it was specifically induced following CWMV infection (S5D Fig). Similarly, we identified 15 copies of the *TaRD21*-like gene and constructed a maximum likelihood phylogenetic tree via MEGA 7 (S5E Fig). TraesCS2B02G540100 was identified as TaRD21 with 59.02% amino acid sequence identity with NbRD21, and the Papain family cysteine protease domain had 74.07% similarity (S5F Fig). Structural homology searches conducted between NbRD21 and three TaRD21 proteins revealed that the three TaRD21 proteins share a similar structure with NbRD21 (S5G Fig). To further investigate the role of TaPIRIN in CWMV infection in wheat, we analyzed the expression pattern of TaPIRIN in response to CWMV infection and found that TaPIRIN was upregulated after CWMV infection (S6A Fig).

**Fig 6:**
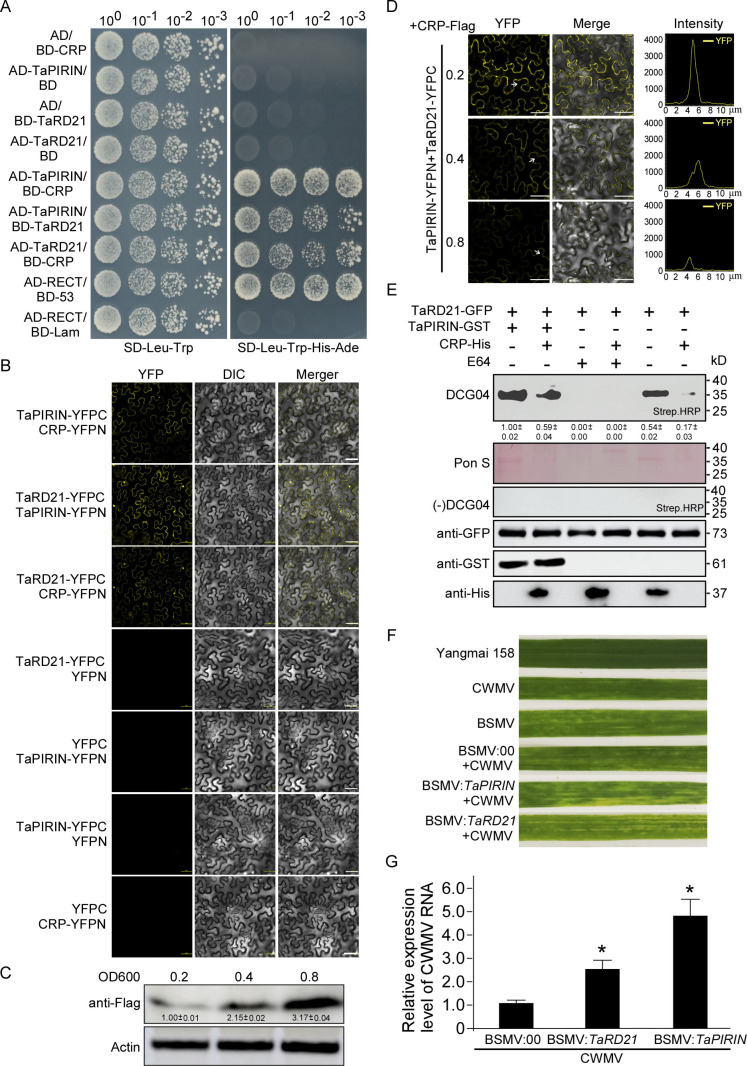
TaPIRIN promote the hydrolase activities of TaRD21 to inhibit CWMV infection. **(A)** Yeast two hybrid assay of showing positive interaction between TaPIRIN and CRP, TaRD21 and CRP, or TaPIRIN and TaRD21. The AD- and BD- plasmids were co-transformed into yeast AH109, and transformants were plated on SD-Leu-Trp for 3 days and transfer to selective SD-Leu-Trp-His-Ade for 5 days. AD-RECT and BD-53 were co-transformed as positive control, the AD-RECT and BD-lam were co-transformed as negative control. **(B)** BiFC assays confirming the interaction of TaPIRIN with CRP, TaRD21 and CRP, or TaPIRIN and TaRD21. TaPIRIN-YFPC or TaRD21-YFPC was agro-injected together with CRP-YFPN into *N. benthamiana* leaves, TaPIRIN-YFPN was agro-injected together with TaRD21-YFPC into *N. benthamiana* leaves and the samples were imaged by confocal microscopy at 48 hpi. TaPIRIN-YFPC agro-injected together with YFPN, TaPIRIN-YFPN agro-injected together with YFPC, TaRD21-YFPC agro-injected together with YFPN and CRP-YFPN agro-injected together with YFPC were shown as negative controls. Scale bar = 25 µm. Each experiment was repeated three times with similar results. **(C)** Western blot analysis of CRP-Flag accumulation in the samples that mentioned in (D) using an anti-Flag specific antibody. The Actin is used to show sample loadings. The numbers below the bands mean the intensity ratio of the bands that calculated by Image J software. **(D)** BiFC assays confirming the interactions of TaPIRIN with TaRD21. TaPIRIN-YFPC was agro-injected together with TaRD21-YFPN and different concentrations of CRP-Flag into *N. benthamiana* leaves, and the samples were imaged by confocal microscopy at 48 hpi. The intensity indicates the strength of interaction. Scale bar = 25 µm. Each experiment was repeated three times with similar results. **(E)** The TaRD21-GFP was purified from apoplastic fluid that extracted from the leaves of *N. benthamiana* plants transiently expressing TaRD21-GFP. TaRD21-GFP was labeled with DCG-04 or not in presence or absence of CRP-His, NbPIRIN-GST or E64, and detected with streptavidin-HRP. Proteins load were analyzed by Western blotting with anti-GFP antibodies (HT801, TransGen Biotech, China), anti-His antibodies (HT501, TransGen Biotech, China) and anti-GST antibodies (HT601, TransGen Biotech, China). The numbers below the bands mean the intensity ratio of the bands that calculated by Image J software. **(F)** Images of leaves taken from Yangmai 158 or CWMV-inoculated or BSMV-inoculated wheat plant or CWMV-inoculated *TaPIRIN*-silenced (BSMV:*TaPIRIN*) or *TaRD21*-silenced (BSMV:*TaRD21*) or non-silenced (BSMV:00) wheat plants at 28 dpi. **(G)** Quantitative RT-PCR analysis of CWMV RNA accumulation in the CWMV-inoculated *TaPIRIN*-silenced, *TaRD21*-silenced or non-silenced wheat plants. Asterisk indicates a significant difference between the CWMV-inoculated *TaPIRIN*-silenced, *TaRD21*-silenced or non-silenced wheat plants (*, **P** ≤ 0.05 by Student’s t-test).

To confirm the interaction between TaPIRIN, TaRD21 and CRP, AD-TaPIRIN and BD-CRP plasmids were used in a YTH assay. The results showed that AD-TaPIRIN interacts with BD-CRP, AD-TaPIRIN interacts with BD-TaRD21, and AD-TaRD21 interacts with BD-CRP in yeast cells (Fig 6A). We further conducted a bimolecular fluorescence complementation (BiFC) assay and confirmed that TaPIRIN interacts with CRP, TaPIRIN interacts with TaRD21, and TaRD21 interacts with CRP (Fig 6B). Since CRP interacts with both TaPIRIN and TaRD21, we suspected that CRP affects the TaPIRIN interaction with TaRD21. To confirm our suspicions, we conducted a bimolecular fluorescence complementation (BiFC) assay. TaRD21-YFPN, TaPIRIN-YFPC and CRP-Flag were transiently coexpressed in *N. benthamiana* leaves. As the concentration of *Agrobacterium* culture carrying CRP-Flag was increased from OD600 = 0.2 to 0.8, the intensity of YFP fluorescence in plant cells was reduced in a dose-dependent manner (Fig 6C and 6D). We further conducted a protease activity profiling assay to probe the effects of CRP and TaPIRIN on TaRD21 protease activity. TaRD21-GFP was transiently overexpressed in *N. benthamiana* leaves. Protein extracts from the leaves were combined with DCG-04 and incubated with or without recombinant CRP or TaPIRIN. The binding of DCG-04 was fully inhibited by treatment with excess E-64 prior to labeling. After 60 min of incubation with DCG-04, one major band corresponding to the protease was identified on the blots using streptavidin-conjugated horseradish peroxidase (HRP). The samples incubated with TaPIRIN presented the strongest band, and the samples incubated with CRP presented the weakest band. The samples incubated with both TaPIRIN and CRP presented a moderate-strength band similar to that of the TaRD21-GFP samples incubated without CRP or TaPIRIN (Fig 6E). In addition, we investigated the effects of *TaPIRIN* and *TaRD21* on CWMV infection. Yangmai 158 seedlings were inoculated with CWMV RNA transcripts, BSMV:00, mixtures of CWMV and BSMV:00, CWMV and BSMV:*TaPIRIN* transcripts, or CWMV and BSMV:*TaRD21* transcripts. At 7 dpi, the infections of CWMV and BSMV were confirmed via RT‒PCR via primers specific for the CWMV *CP* or BSMV *CP* genes (S6B and S6C Fig), and the expression levels of *TaPIRIN* and *TaRD21* in these plants were determined via qRT‒PCR (S6D and S6E Fig). By 14 dpi, the plants inoculated with CWMV or BSMV transcripts presented mosaic symptoms. The plants inoculated with the CWMV and BSMV:*TaPIRIN* mixed transcripts or with the CWMV and BSMV:*TaRD21* mixed transcripts presented stronger mosaic symptoms than those inoculated with the CWMV and BSMV:00 mixed transcripts did (Fig 6F). CWMV genomic RNA accumulation revealed that stronger symptoms were associated with significantly increased accumulation of CWMV CP (Fig 6G).

## Discussion

PIRIN proteins belong to the cupin domain-containing superfamily and are highly conserved among mammals, plants, fungi and prokaryotic organisms [1]. Several types of activities and protein interactions have been assigned to PRN proteins in various organisms, including humans, bacteria, and several plant species. Early studies revealed that AtPirin1 is involved in abscisic acid (ABA)-mediated germination and early seedling development by interacting with the heterotrimeric Gα subunit AtGPA1 [3], and AtPirin2 was found to play a role in lignin metabolism by suppressing the accumulation of S-type lignin in stems [7]. *Saccharopolyspora* spinose pirin-like protein is involved in strain growth and spinosad biosynthesis [44]. In this study, we identified a *Nicotiana benthamiana NbPIRIN* and found that RNAi-mediated silencing of *NbPIRIN* inhibited vegetative growth, and overexpression of *NbPIRIN* promoted vegetative growth (Fig S1). These results indicated that the pirin-like protein may have similar roles in vegetative growth among different species. Here, we showed that NbPIRIN is important for *N. benthamiana* resistance to Chinese wheat mosaic virus (CWMV) in addition to vegetative growth. Since RNAi*PIR*#1, RNAi*PIR*#2 and RNAi*PIR*#7 plants accumulate more CWMV RNA than wild-type plants, and the *NbPIRIN* transgenic lines accumulate less CWMV RNA than wild-type plants (Fig 1).

Studies showed that the Arabidopsis PIRIN2 interacts with three closely related PLCPs, XCP2, RD21A and RD21B and PIRIN2 increases protease activities of XCP2 and RD21A [6]. The yeast two-hybrid and BiFC experiments demonstrated that NbPIRIN interacts with NbRD21. Intriguingly, the protease activity of NbRD21 increased when the NbPIRIN protein was present, as determined by analysis of the amount of mature 25–35 kDa active protease (Fig 2). These results suggested that NbPIRIN has similar functions with AtPIRIN2 on interact with PLCPs and increases protease activities of PLCPs. The XCP2 activity declined during the time course, but that the decline was suppressed in samples pretreated with PIRIN2, and the XCP2 activity was suppressed in the presence of PIRIN2 at the start of the time course, but recovered to the same or somewhat higher activity level at the end of the time course experiment, indicating that PIRIN2 inhibits XCP2 activity, but the inhibition is reversible and eventually results in stabilization of XCP2 [6]. In our study, no effect on stabilization of NbRD21 was found in the presence of NbPIRIN (Fig S2), indicating that PIRIN protein may have different functions among different species. PLCPs possess a conserved catalytic triad composed of three amino acid residues: Cys, His, and Asp. Our data indicate that mutation of the key enzyme activity sites of NbRD21 reduces its resistance to CWMV infection, indicating that the protease activity of NbRD21 is important for resistance to CWMV (Fig 2). Thus, we propose that NbPIRIN could function as a scaffolding protein for PLCPs, allowing high proteolytic activity whenever needed.

No other evidence for a specific role of NbRD21 in plant pathogenesis has been published. However, other PLCPs have well-documented functions in plant pathogenesis. For example, the PLCP RCR3 is required for Cf-2-dependent resistance to *Cladosporium fulvum* and contributes to resistance to *Phytophthora infestans* in tomato [27,45]. PLCP C14 contributes to the defense against *P. infestans* in *N. benthamiana* [16,17]. PLCP RD19 is required for RRS1-R-mediated resistance to *R. solanacearum* [28], and RD21A mutants are more susceptible to *Botrytis* in Arabidopsis [18]. PLCP CCP1 modulates the salicylic acid-mediated defense against sugarcane mosaic virus (SCMV) infection in maize [33]. Most importantly, in most previously reported cases, PLCPs have been shown to increase resistance to invading pathogens. Our findings revealed that silencing NbRD21 expression in *N. benthamiana* markedly promoted CWMV infection and that overexpressing NbRD21 in *N. benthamiana* significantly suppressed CWMV infection (Fig 3). Given the resistance roles of PLCPs in plants infected by fungi, bacteria or viruses, PLCP-mediated resistance against diverse biotic stresses may be conserved.

**Fig 7:**
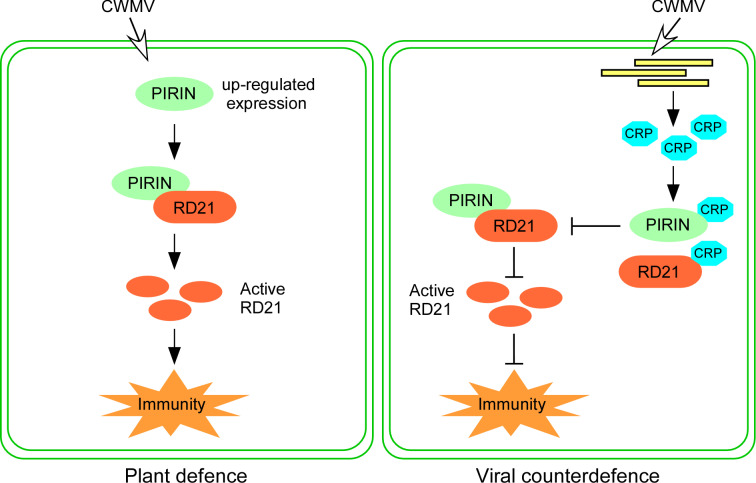
Functions of PIRIN and RD21 during plant immune-signaling. PIRIN interact with RD21 to promote the PLCP activity activating plant immunity. The expression level of PIRIN was up-regulated when plants infected with CWMV. PIRIN interact with RD21 and increase its protease activities to active plant immunity (plant defence). CWMV CRP interact with both PIRIN and RD21 interfering the interaction between PIRIN and RD21 to inhibit the protease activities of RD21 and then inhibit plant immunity (Viral counterdefence).

For infection to be compatible, plant PLCPs need to be inhibited by pathogen effectors [32]. Ample evidence reveals that various pathogens, including fungi, oomycetes, bacteria and viruses, have evolved to produce effectors that hinder PLCP activities, thus assisting their infection in plants [15,17,31,33,46]. A common tactic is the suppression of PLCP activities, often achieved through the secretion of effectors or the deployment of endogenous inhibitors to stifle host defenses [33]. For example, the oomycete pathogen *P. infestans* has been shown to secrete the cystatin-like effectors EPIC1 and EPIC2B to inhibit the function of the C14 protease in tomato and potato [16]. In the fungal maize pathogen *Ustilago maydis*, the effector protein Pit2 functions as a substrate mimic, releasing an inhibitory peptide upon cleavage by apoplastic PLCPs [47]. In citrus plants, Sec-delivered effector 1 (SDE1) from Huanglongbing-associated bacteria has been verified to inhibit the functions of immune-related PLCPs [31]. In a geminivirus, tomato yellow leaf curl virus (TYLCV), the V2 protein was reported to interact with the tomato PLCP CYP1 and suppress its activity [48]. A WYMV-encoded Nia protein suppresses TaRD21A activity to promote viral infection [29]. Nia-Pro of SCMV targets corn cysteine protease 1 (CCP1) to undermine salicylic acid-mediated defense in maize [33]. In our investigation, we found that CWMV CRP, but not the other CWMV proteins, interacted with both NbPIRIN and NbRD21 via yeast two-hybrid assays and BiFC and GST pulldown assays (Fig 4). CRP is a 19 kDa cysteine-rich RNA silencing suppressor and has been identified as a VSR protein that inhibits the spread of silencing signals [37]. In addition, we found that CWMV CRP constrains NbRD21 protease activity by interfering with the interaction between NbPIRIN and NbRD21 (Fig 5). While PLCPs are targeted by specific effectors, the inhibiting effectors do not possess conserved motifs, indicating that these effectors have evolved independently to disrupt the activity of particular PLCPs [31].CWMV causes yellow mosaic disease in wheat crops in China, and it only infects wheat in the field [36]. In our investigation, we identified homologs of NbPIRIN and NbRD21 in wheat and verified the interactions between TaPIRIN, TaRD21 and CRP via yeast two-hybrid and BiFC assays. In addition, we found that CRP constrains NbRD21 protease activity. We also found that the TaPIRIN protein increased the protease activity of TaRD21 and that CRP decreased the protease activity of TaRD21. Silencing TaPIRIN and TaRD21 expression in wheat markedly promoted CWMV infection (Fig 6). These findings suggest that there is a similar mechanism of CWMV resistance in *N. benthamiana* and wheat. Taken together, our integrated genetic and biochemical studies have delineated an offensive and defensive relationship between plants and viruses in *N. benthamiana* and wheat. Based on these results, we have developed a working model to illustrate how *N. benthamiana* and wheat governs resistance to CWMV and how CWMV breaches the host defence (Fig 7). In this model, CWMV infection triggers the up-regulation of PIRIN. PIRIN interacts with RD21, promoting its protease activity to activate host immunity. In turn, CWMV suppression the host immunity by synthesizing CRP to hijack both PIRIN and RD21, which interfere the interaction between PIRIN and RD21, which further inhibits the protease activity of RD21 resulting suppressive host immunity.

## Materials and Methods

### Stable expression in *N. benthamiana* and growth conditions

All overexpression transgenic, RNAi plants used in this study were generated in *N. benthamiana*. For construction of NbPIRIN or NbRD21 RNA-silencing transgenic lines, a specific NbPIRIN or NbRD21 fragment was selected using a virus-induced gene silencing (VIGS) tool in the Sol Genomics Network (https://www.solgenomics.net/), the specific sequences employed for silencing NbPIRIN and NbRD21 was shown in red in S1 Table. The hpRNA cassette contains the restricted tev movement 1 (RTM1) gene as an intron flanked by this specific fragment in sense and antisense orientations. The expression cassette was digested with BamHI and SacI and inserted into the binary vector pCAMBIA2301 using the same restriction sites to construct the NbPIRIN-, or NbRD21-RNA interfering vector under the control of the 35S promoter and terminated by the NOS terminator. For construction of NbPIRIN or NbRD21 overexpression transgenic lines, the full length of NbPIRIN and NbRD21 was cloned from *N. benthamiana* and cloned into the pCAMBIA1301 vector under the control of the 35S promoter, respectively. The vector for RNAi assay or overexpression assay was then transferred into *A. tumefaciens* LBA4404 for transformation of *N. benthamiana*. Transformation and Basta selection for the different generations of transformants was as described in Forestier et al., 2021 [49]. *A. tumefaciens* LBA4404 containing the vectors of interest was prepared and added to the co-cultivation solution. Infected leaf discs were incubated on co-cultivated for 3–4 days on a medium with 0.1 mg/L of 1-naphthaleneacetic acid (NAA) and 1 mg/L of 6-benzylaminopurine (BAP) (Sigma-Aldrich, Burlington, MA, USA), then transferred to selection medium with 5 mg/L of glufosinate and 500 mg/L of cefotaxime (Sigma-Aldrich). Shoots appeared after 30–40 days and were transferred to rooting medium (2.65 g/L of modified MS n◦4 M0238 from Duchefa, 825 mg/L of NH4NO3, 30 g/L of sucrose, 100 mg/L of myo-inositol, 0.5 mg/L of the same 3 vitamins as the co-cultivation medium, KOH to adjust the pH to 5.7–5.8, and 6 g/L of agar) with doubled concentration of glufosinate. Roots were developed 15–30 days after transfer, allowing the transformed seedlings to be put into the same compost used for WT plants. All overexpression and RNAi plants or wild type *N. benthamiana* were grown in a growth chamber set at 25±2°C with 70% relative humidity and a 16 h light and 8 h dark photoperiod. The wheat plants were cultivated in soil under normal growth conditions in a growth chamber (16h light and 8h dark cycle at 16±2°C, 70% relative e humidity).

### CWMV inoculation

Plasmid pCB-35S-RNA1 and pCB-35S-RNA2, containing full-length CWMV RNA1 or RNA2 sequence that constructed by Yang et al., 2016 [40], were individually transformed into *A. tumefaciens* strain GV3101. The Agrobacterium cultures were grown overnight, pelleted through centrifugation, and then incubated for 2 hours in an infiltration buffer (10 mM MES, pH 5.6, 10 mM MgCl2, and 150 μM acetosyringone). Agrobacterium culture carrying pCB-35S-RNA1 (OD600 = 0.6–0.8) was mixed with an equal volume of Agrobacterium culture carrying pCB-35S-RNA2. The mixed cultures were infiltrated individually into leaves of *N. benthamiana* plants using needleless syringes. Inoculation of wheat seedlings with in vitro transcribed CWMV RNAs was as reported previously [50]. Briefly, plasmids were individually linearized with SpeI restriction enzyme followed by in vitro transcription using the Ambion Message Machine kit (Invitrogen). In vitro transcribed CWMV RNA1 transcript (5 μg) was mixed with an equal amount of transcript representing CWMV RNA2. The mixed RNA transcripts were individually diluted in 1× FES buffer (0.06 M K2HPO4, 0.1 M glycine, 1% tetrasodium pyrophosphate, 1% celite, and 1% bentonite in nuclease-free water, pH 8.5) followed by rub-inoculation to the second true leaf of each wheat seedling (10 μL per leaf). All experiments were repeated at least 3 times, and each experiment included at least 6 plants. CWMV infection rate is 100%. Symptoms in *N. benthamiana* or wheat plants infected with the CWMV were photographed at 21 dpi. The CWMV infiltrated plants were then grown inside a climatic incubator at 15°C, 65±5% relative humidity, and a 16-hour light/8-hour dark photoperiod.

### Plasmid construction

The full length of NbPIRIN and NbRD21 were cloned from cDNA of *N. benthamiana*. NbPIRIN cloned into the pGWB336-35S-RFP, pGEX4T-2, pGADT7, pGTQL122-35S-YFPC vectors, respectively. NbRD21 cloned into the pGWB505-35S-GFP, pGWB336-35S-RFP, pGBKT7, pGTQL122-35S-YFPC, pGTQL121-35S-YFPN vectors, respectively. The full length of CWMV CRP was cloned from Plasmid pCB-35S-RNA2 and cloned into the pGWB505-35S-GFP, pET-32a, pGBKT7, pGTQL121-35S-YFPN vectors, respectively. The NbRD21^CHN^ (C161A, H297A, and N317A) sequence were generated by piecewise amplification from pGBKT7-NbRD21 and recombined through overlapping PCR using plasmid pGWB505-35S-GFP as the template. The full length of TaPIRIN and TaRD21 were cloned from cDNA of wheat cv. Yangmai 158 plants. TaPIRIN was cloned into the pGEX4T-2, pGADT7, pGTQL121-35S-YFPN vectors, respectively. TaRD21 cloned into the pGWB505-35S-GFP, pGBKT7, pGTQL122-35S-YFPC, pGWB511-35S-flag vectors, respectively. All the gene sequence was amplified using 2 × Phanta UniFi Master Mix (P526, Vazyme Biotech Co., Ltd., China). All primers used in this study are listed in S3 Table.

### Yeast two hybrid assay

To investigate the interaction between CRP and NbPIRIN, CRP and NbRD21, NbPIRIN and NbRD21, TaPIRIN and CRP, TaPIRIN and TaRD21, respectively. The bait constructs were generated by cloning CRP, NbRD21 and TaRD21 full-length cDNAs into pGBKT7, respectively. The cDNAs of NbPIRIN, TaPIRIN and TaRD21 were cloned into pGADT7 as the prey constructs (see primers in S3 Table). The resulting prey and bait constructs were confirmed by sequencing analysis and transformed in pairs into yeast strain AH109 as the description of BD library construction & screening kit (Clontech, USA). The co-transformed cells were grown on the SD/-Leu/-Trp medium for 3 days and then on the SD/-Trp/-Leu/-His/-Ade medium for 5 days. Yeast cells co-transformed with AD-RECT+BD-Lam was used as negative control and AD-RECT+BD-53 was used as positive controls.

### Bimolecular fluorescence complementation (BiFC) assay

NbRD21, CRP and TaPIRIN were cloned into pGTQL121-35S-YFPN vectors, NbPIRIN, TaPIRIN and TaRD21 were cloned into pGTQL122-35S-YFPC. All the recombinant vector were individually transformed into *A. tumefaciens* strain GV3101. The Agrobacterium cultures were grown overnight, pelleted through centrifugation, and then incubated for 2 hours in an infiltration buffer (10 mM MES, pH 5.6, 10 mM MgCl2, and 150 μM acetosyringone). Agrobacterium culture carrying P35S:NbPIRIN-YFPC (OD600 = 0.6–0.8) was mixed with an equal volume of Agrobacterium culture P35S:CRP-YFPN. Agrobacterium culture carrying P35S:NbPIRIN-YFPC, P35S:NbRD21-YFPN, P35S:NbRD21-YFPC, P35S:CRP-YFPN, P35S:TaPIRIN-YFPC, P35S:CRP-YFPN, P35S:TaPIRIN-YFPN and P35S:TaRD21-YFPC were mix in the same way as expected. The mixed cultures were infiltrated individually into leaves of *N. benthamiana* plants using needleless syringes. At 60 h post agroinfiltration, the infiltrated leaves were harvested and examined under a confocal microscope. Co-expressed empty vector and one recombinant vector were used as controls. For competitive binding experiment, CRP was cloned into pGWB511-35S-Flag vectors and then transformed into *A. tumefaciens* strain GV3101. The mixed Agrobacterium culture carrying P35S:NbPIRIN-YFPC and P35S:NbRD21-YFPN (OD600 = 0.6–0.8) was then mixed with an equal volume of Agrobacterium culture carrying P:35S-CRP-Flag (OD600 = 0.4, 0.6 and 1.6), respectively. The mixed cultures were infiltrated individually into leaves of *N. benthamiana* plants using needleless syringes. At 48 h post agroinfiltration, the infiltrated leaves were harvested and examined under a confocal microscope. Fluorescence intensity indicates the intensity of interaction.

### Confocal microscopy

To investigate the subcellular localization patterns of CRP, NbRD21 and NbPIRIN, NbRD21 and NbPIRIN fused to the C-terminus of red fluorescent protein (NbRD21-RFP, NbPIRIN-RFP), CRP and NbRD21 fused to the C-terminus of green fluorescent protein (CRP-GFP, NbRD21-GFP) were obtained, and infiltrated individually or together into *N. benthamiana* leaves. At 48 h post infiltration, the infiltrated leaves were harvested and examined under a confocal microscope (Nikon, Tokyo, Japan; A1+A1R) for green and red fluorescence. GFP was excited at the wavelength of 488 nm and the emission was captured at 500–550 nm. RFP was excited at 561 nm and the emission was captured at 575–620 nm.

### RNA extraction and quantitative reverse transcription polymerase chain reaction (qRT-PCR)

Total RNA was extracted from *N. benthamiana* or wheat tissue samples using the HiPure plant RNA mini kit (Magen, Guangzhou, China). First-strand cDNAs were synthesized using ran-dom primers, 1 μg total RNA per 20 μL reaction, and the First Strand cDNA Synthesis Kit (TOYOBO, Osaka, Japan). Quantitative PCR was carried out using the SYBR Green qRT-PCR kit (Vazyme, Nanjing, China) with CWMV-CP (S1F/S1R in S3 Table) primers and NbUBC (S22F/S22R in S3 Table) on an Applied Biosystems QuantStudio 6 Flex system (Applied Biosystems, Foster City, CA, USA). Relative expressions of the assayed genes were calculated using the 2 -ΔΔCt method. Each treatment has three biological replicates with three technical replicates each. Each experiment was repeated three times. The primers used in this study are listed in the S3 Table.

### Western blot assay

For total protein extraction, *N. benthamiana* tissue samples were homogenized individually in a lysis buffer containing 0.5% SDS, 50 mM Tris-HCl (pH 8.0), 10 mM EDTA, 0.5 M sucrose, 1 mM MgCl_2_ and 5 mM DTT. Protein samples were analyzed in SDS-PAGE gels through electrophoresis, and then transferred onto nitrocellulose membranes. The blots were incubated in a blocking buffer (5% skim milk and 0.05% Tween 20 in 1×PBS) for 120 min followed by detection using specific anti-flag (AE092, ABclonal Technology, China), anti-GFP (HT801, TransGen Biotech, China) and anti-CWMV CP antibodies (Laboratory preparation) and then an HRP-conjugated anti-mouse (HS002, TransGen Biotech, China) or anti-rabbit (HS001, TransGen Biotech, China) secondary anti-body. Detection signal was visualized using an Amersham Imager 680 machine (GE Healthcare BioSciences, Pittsburgh, PA, USA).

### Pull-down assays

For protein production in Escherichia coli, the full length of NbPIRIN and NbRD21 were inserted into the pGEX4T-2 vector and the full length of CRP was inserted into the pET-32a vector. The resultant plasmid DNA was transformed into Rosetta 2 (DE3) cells (Novagen, Madison, WI). Protein expression was induced for 8 h at 25°C after the addition of isopropy-β-D-thiogalactoside (IPTG) to a final concentration of 0.1 mM. Cells were collected by centrifugation, washed, and stored at -70°C. To extract proteins, cells were suspended in lysis buffer containing 0.5 mM EDTA, 1% Triton, 20 mM Tris-HCl, 0.15 M NaCl, 1 mM DTT and protease inhibitors (1 mM PMSF) and ultrasonic wall-breaking under frequency: 100–200 w 2 min, pause 4 s run 2 s. Samples were centrifuged at 13,000 rpm for 15 min at 4°C and the supernatants were collected.

For GST-tag protein purification, supernatants were mixed with glutathione-Sepharose resin (Amersham Pharmacia, Piscataway, NJ) for 2 h, centrifuged at 500 rpm for 2 min at 4°C, washed with Tris-NaCl buffer (1 mM PMSF, 1% Triton, 50 mM Tris-HCl, 100 mM NaCl) and the proteins were eluted with 15 mM glutathione. Proteins were also verified by SDS-PAGE analysis and Western blotting with the anti-GST antibody (TransGen Biotech, Beijing, China).

For His-tag protein purification, supernatants were mixed with BeyoMag IDA-Ni Magnetic Agarose Beads (Beyotime, Shanghai, China) for 1 h, place on magnetic rack (FMS012/FMS024) for 10 seconds to remove supernatant at 4°C, washed with Binding/Wash Buffer (10 mM Tris, 500 mM NaCl, pH7.4) and the proteins were eluted with Elution Buffer (10 mM Tris, 500 mM NaCl, 500 mM Imidazole, pH7.4). Proteins were also verified by SDS-PAGE analysis and Western blotting with the anti-His antibody (HT501, TransGen Biotech, China).

For binding, 50 μl of the purified NbPIRIN-GST extract were added to glutathione-Sepharose resin washed with Tris-NaCl buffer. GST protein was used as a control. The purified CRP-His was added to resin and incubated for 2 h with gentle rotation at 4°C, precipitated, and washed three times with Tris-NaCl buffer. SDS-containing gel loading buffer (100 μl) was added to the resin before a brief boiling. Samples (4 μl) were analyzed by SDS-PAGE electrophoresis and Western blotting using anti-His and anti-GST antibodies (HT601, TransGen Biotech, China).

### Apoplastic fluid (AF) isolation

To prepare AF, leaves from *N. benthamiana* plants that transiently overexpressed with NbRD21-GFP, NbRD21CHN-GFP or TaRD21-GFP by agroinfiltration were submerged in water within a vacuum chamber for 30 min at 400 mbar. Subsequently, these drained leaves were carefully positioned inside the barrel of a 50 mL syringe, aligning the leaf edges with the barrel ends. The barrel, with the needle hub pointing downward, was then placed into a 50 mL centrifuge tube. This assembly was subjected to centrifugation for 20 min at 2000 ×g and 4 °C. Following centrifugation, the resulting AF was extracted from the tube, then the AF was mixed with GFP-Trap magnetic agarose (gtma-20, Proteintech, USA) for 2 h at 4°C, place on magnetic rack (FMS012/FMS024) for 10 seconds to remove supernatant, washed with Wash Buffer (1 mM DTT, 30% Glycerol, 4 M Guanidine HCl, 500 mM NaCl, 2% Nonidet P40 Substitute, 1% SDS, 0.2 mM TCEP, 1% Triton X-100, 8 M Urea) and the proteins were eluted with Elution Buffer (0.2 M glycine, pH2.5) and neutralized with 0.1 volume of 1 M Tris base and promptly stored at −20 °C.

### Protease activity profiling

To investigate NbRD21 activity, the NbRD21-GFP and NbRD21^CHN^-GFP were purified from AF that extracted from the leaves of *N. benthamiana* plants transiently expressing NbRD21-GFP or NbRD21^CHN^-GFP. NbRD21-GFP and NbRD21^CHN^-GFP were labeled in a solution consisting of 2 μM DCG-04, 50 mM sodium acetate (pH 5.5), and 1 mM dithiothreitol (DTT) at room temperature. The labeling reactions were stopped by adding 10 μl 5× SDS-PAGE sample buffer. Proteins in all samples were separated by SDS-PAGE, transferred onto nitrocellulose membrane (Bio-Rad, www.biorad.com), and detected with streptavidin-HRP (1:3000; Ultrasensitive; Sigma, www.sigmaaldrich.com).

### Activity-based protein profiling (ABPP) assay

The NbRD21-GFP, NbRD21^CHN^-GFP and TaRD21-GFP were purified from AF that extracted from the leaves of *N. benthamiana* plants transiently expressing NbRD21-GFP, NbRD21^CHN^-GFP or TaRD21-GFP. For in vitro assay, the concentration of purified NbRD21-GFP, NbRD21^CHN^-GFP and TaRD21-GFP was adjusted to 0.2 mg ml^−1^ with 15 mM sodium phosphate buffer, pH 6.0, 0.2 mM DTT and then preincubated with 5 μM E-64 (Sigma-Aldrich, Shanghai, China) in a total volume of 200 μL for 60 min at room temperature prior to the addition of 0.2 μL of 2 mM DCG-04. For inhibition assays, purified proteins were incubated together with 0.5 μM purified CRP protein and then treated with 2 μM DCG-04 for 60 min in presence or absence of E-64. For enhance assays, purified proteins were incubated together with 0.5 μM purified PIRIN protein and then treated with 2 μM DCG-04 for 60 min in presence or absence of E-64. The reactions were stopped by the addition of 10 μL of 5× SDS-PAGE sample buffer. The resulting samples were analyzed via SDS-PAGE. Proteins attached to the nitrocellulose membranes were detected using streptavidin-conjugated horseradish peroxidase (HRP; 1:3000, Abbkine Scientific Co, California, USA, Cat. No. A21000). As fluorescent intensity of bound probe reflects activity the signal was quantified using ImageJ Software.

### Virus-induced gene silencing

Barley stripe mosaic virus (BSMV)-based VIGS vector (pBSMV) was used to silence gene expressions in wheat. A 200-bp fragment representing the partial sequence of *TaPIRIN* and *Ta*RD21 was amplified through RT-PCR using primers listed in the S3 Table. The PCR fragment was cloned, in the sense orientation, into the pBSMV vector (provided by Professor Zhensheng Kang, Northwest Agricultural and Forestry University, Yangling, Shaanxi, China) to generate pBSMV:*TaPIRIN* and pBSMV:*TaRD21*. All plasmids were propagated in *E. coli* DH5α cells, and sequenced before use. BSMV RNAs were individually transcribed *in vitro* from linearized plasmid DNAs (pBSMVα, pBSMVβ, pBSMVγ, pBSMV:*TaPIRIN* and pBSMV:*TaRD21*, respectively) using the RiboMAX Large Scale RNA Production System T7 *in vitro* transcription kits instructed by the manufacturer (P1300, promega, USA). The resulting BSMVα and BSMVβ transcripts were mixed with BSMVγ, BSMV:*TaPIRIN* or pBSMV:*TaRD21* transcripts to produce BSMB:00 (empty vector), BSMV:*TaPIRIN* and pBSMV:*TaRD21*, respectively. The mixed transcripts were individually diluted 20 times with RNase-free water. The diluted transcripts (0.5 μLper virus) were further resuspended in 9 μL inoculation buffer (1 mL 50 mM glycine and 50 mM K_2_HPO_4_, pH 9.2), and rub-inoculated to the youngest leaf of a 2-leaf stage wheat seedling. Sixteen plants were inoculated for each treatment. Wheat seedlings inoculated with buffer only were used as mock-infected controls. The inoculated seedlings were grown inside a dark growth chamber at 28 °C for 24 h, and then in a chamber set at 28 °C and a 16h light/8h dark photoperiod.

## Supporting information

S1 Table Gene unique identifiers and sequence. (DOCX)

S2 Table The total of 25 positive clones obtained from the Y2H screening with NbPIRIN as bait. (DOCX)

S3 Table Detail information of primers. (DOCX)

S1 Fig (A) Quantitative RT-PCR analysis of *NbPIRIN* relative expression level in wild type and three *NbPIRIN* silencing lines. An asterisk above the bar indicates a significant difference between the two treatments (*, P ≤ 0.05 by Student’s t-test). **(B)** Vegetative growth of wild type and three *NbPIRIN* silencing lines at 4 weeks and 8 weeks. **(C)** Quantitative RT-PCR analysis of *NbPIRIN* relative expression level in wild type and three *NbPIRIN*-overexpressing transgenic lines. An asterisk above the bar indicates a significant difference between the two treatments (*, P ≤ 0.05 by Student’s t-test). **(D)** Vegetative growth of wild type and three *NbPIRIN*-overexpressing transgenic lines at 4 weeks and 8 weeks. **(E)** Photograph of the whole plants of WT, RNAi*PIR*#1, RNAi*PIR*#2 and RNAi*PIR*#7 at the age of inoculation. **(F)** Photograph of WT, RNAi*PIR*#1, RNAi*PIR*#2 and RNAi*PIR*#7 plant leaves after 21 days post inoculation with agroinfiltration GV3101. **(G)** Photograph of the whole plants of WT, OE*PIR*#4, OE*PIR*#7 and OE*PIR*#12 at the age of inoculation. **(H)** Photograph of WT, OE*PIR*#4, OE*PIR*#7 and OE*PIR*#12 plant leaves after 21 days post inoculation with agroinfiltration GV3101. **(I)** Quantitative RT-PCR analysis of *NbPIRIN* relative expression level in the CWMV-infected and uninfected wheat leaves. An asterisk above the bar indicates a significant difference between the two treatments (*, P ≤ 0.05 by Student’s t-test). **(J)** Quantitative RT-PCR analysis of *NbPIRIN* relative expression level in wild type, three *NbPIRIN* silencing lines and three *NbPIRIN*-overexpressing transgenic lines. An asterisk above the bar indicates a significant difference between the two treatments (*, P ≤ 0.05 by Student’s t-test).
(TIF)

S2 Fig (A) A phylogenetic tree of PLCPs in Arabidopsis and *N. benthamiana.
* Each group is represented by a different colour. Stars represent NbRD21. First, the NbRD21 protein sequence is retrieved in the phylogenetic genome using Blastp with evalue =1E-7. Phylogenetic analysis is performed with phylosuite software after integration of Arabidopsis data, and finally visualization is performed with Figtree software. **(B)** Bar plot of the intensity ratio of the bands in Fig 2F calculated by Image J software. There were three biological replicates of each treatment. An asterisk above the bar indicates a significant difference between the two treatments (*, P ≤ 0.05 by Student’s t-test). **(C)** Bar plot of the intensity ratio of the bands in Fig 2G calculated by Image J software. Experiment in Fig 2G was repeated three times, and the intensity ratio was obtained according to three repeated calculations. An asterisk above the bar indicates a significant difference between the two treatments (*, P ≤ 0.05 by Student’s t-test). **(D)** Western blot analysis of NbRD21-GFP using a GFP specific antibody at different times after the addition of the protein translation inhibitor cycloheximide (CHX) and adenosine triphosphate (ATP), Protein load of GST and PIRIN-GST were analyzed by Western blotting with anti-GST antibodies (HT601, TransGen Biotech, China).
(TIF)

S3 Fig (A) Quantitative RT-PCR analysis of *NbRD21* relative expression level in wild type, *NbRD21* silencing lines and *NbRD21*-overexpressing transgenic lines. An asterisk above the bar indicates a significant difference between the two treatments (*, P ≤ 0.05 by Student’s t-test). **(B)** Vegetative growth of wild type, *NbRD21* silencing lines and *NbRD21*-overexpressing transgenic lines at 4 weeks and 8 weeks. **(C)** Photograph of the whole plants of WT, RNAi*RD21*#1, RNAi*RD21*#5, OE*RD21*#1 and OE*RD21*#6 at the age of inoculation. **(D)** Photograph of WT, RNAi*RD21*#1, RNAi*RD21*#5, OE*RD21*#1 and OE*RD21*#6 plant leaves after 21 days post inoculation with agroinfiltration GV3101. **(E)** Quantitative RT-PCR analysis of *NbRD21* relative expression level in WT, RNAi*RD21*#1, RNAi*RD21*#5, OE*RD21*#1 and OE*RD21*#6. An asterisk above the bar indicates a significant difference between the two treatments (*, P ≤ 0.05 by Student’s t-test).
(TIF)

S4 Fig Western blot analysis of CRP-GFP using a GFP specific antibody. Protein load of RFP and NbRD21-RFP were analyzed by Western blotting with anti-RFP antibodies (6g6, Chromoteck, USA).
(TIF)

S5 Fig (A) Phylogenetic analysis of the PIRIN proteins from *Triticum aestivum* and *N. benthamiana* constructed by the neighbor-joining method in MEGA-7. (B) Full length PIRIN amino acid sequences from *T. aestivum* and *N. benthamiana* were aligned using Bioedit software. The overlined areas represent the PIRIN domain, and the numbers represent homology. **(C)** Structural homology searches made between NbPIRIN and three TaPIRIN proteins showed that three TaPIRIN have similar protein structure to NbPIRIN. **(D)** Quantitative RT-PCR analysis of TraesCS1A02G391900, TraesCS1B02G420000 and TraesCS1D02G40000 in the CWMV-infected and uninfected wheat leaves. An asterisk above the bar indicates a significant difference between the two treatments (*, P ≤ 0.05 by Student’s t-test). **(E)** Phylogenetic analysis of the RD21 proteins from *T. aestivum* and *N. benthamiana* constructed by the neighbor-joining method in MEGA-7. **(F)** Full length RD21 amino acid sequences from *T. aestivum* and *N. benthamiana* were aligned using Bioedit software. The overlined areas represent the Papain family cysteine protease domain, and the numbers represent homology. **(G)** Structural homology searches made between NbRD21 and three TaRD21 proteins showed that three TaRD21 have similar protein structure to NbRD21.
(TIF)

S6 Fig Analyses of BSMV and CWMV infection in VIGS vector-inoculated wheat plants. (A) Quantitative RT-PCR analysis of *TaPIRIN* relative expression level in the CWMV-infected and uninfected wheat leaves. An asterisk above the bar indicates a significant difference between the two treatments (*, P ≤ 0.05 by Student’s t-test). **(B)** Confirmation of BSMV infection in the BSMV-inoculated, BSMV:*TaPIRIN* -inoculated or BSMV:*TaRD21* -inoculated wheat plants through RT-PCR at 7 dpi. **(C)** Confirmation of CWMV infection in the CWMV-inoculated BSMV:00 and CWMV-inoculated, BSMV:*TaPIRIN* and CWMV-inoculated, or BSMV:*TaRD21* and CWMV-inoculated wheat plants through RT-PCR at 7 dpi. **(D)** Confirmation of *TaPIRIN* silencing in the BSMV:00 and BSMV:*TaPIRIN* CWMV-inoculated wheat plants through RT-PCR at 7 dpi. **(E)** Confirmation of TaRD21 silencing in the BSMV:00 and BSMV:*TaRD21* CWMV-inoculated wheat plants through RT-PCR at 7 dpi.
(TIF)

S1 Raw Data The raw data of RT-qPCR and intensity ratio of western blot bands in all figures. (XLSX)
