# Supplementary material for: NbPIRIN promotes the protease activity of papain-like cysteine protease NbRD21 to inhibit Chinese wheat mosaic virus infection
Source: PLoS Pathog. 2025 Apr 2;21(4):e1013037. doi: 10.1371/journal.ppat.1013037 (PMC11978040; doi:10.1371/journal.ppat.1013037)
Supplement: S1 Table — (DOCX) [file ppat.1013037.s001.docx]

TableS1.Geneuniqueidentifiers,thecorrespondingdatabaseandsequence.

| Name | Genbanknumber | correspondingdatabase | Sequence(CDS) |
| --- | --- | --- | --- |
| NbPIRIN | PQ728641 | https://www.ncbi.nlm.nih.gov | ATGAGAGCTATTTTTAAACAATTATTAGTGAATTATTTCCCATTTGTTAGAGCTAAAATACTATCAAGAAAGTCAATTTATATTAAAAATATCATGTCTGAATCAAATCAATATGATTCTTGTTTTGATAGACCAAGATTGGTAATTAAGAAAGTTGCGGCTAAGCCTCAAAGTGCAGCGAATGGAGCTGTTGTTAGAAGAAGCATTGGAAGGCCTGAATTGAGGAATCTTGATCCATTCCTCATGTTGGATGAATTTTCAGTTGCGGCTCCTGCTGGATTTCCTGACCATCCACACAGAGGTTTTGAGACAGTAACTTACATGCTAGAGGGAGCTTTTACTCATCAAGATTTTGCTGGTCACAAGGGTACAATCAATACTGGTGATGTGCAGTGGATGACAGCAGGAAGAGGTATAATTCACTCAGAAATGCCTGCAGGAGAAGGCAGTCAAAAGGGGTTGGCACTTTGGATAAATCTTTCTTCTAAGGACAAAATGATTGAGCCAAGGTATCAAGAACTGCTGAATGAGGACATACCAACAGCAGAAAATGAAGGGGTTGAAGTAAAAATCATAGCAGGTGAAGCAATGGGTGTTCAATCTCCAGTTTATACACGAACGCCTACAATGTACCTTGATTTCACCCTACAACCAACATCTTATTATCATCAACCCACCCCTGAGTCTTGGAACGCGTTCGTGTACATAGTTGAAGGAGCAGGCGTATTCGGAATTCCAAATTCAGGTCCTGTATCAGCTCACCATTGCTTGGTTTTAGGCCCTGGAGAAGGACTTAGTGTGTGGAACAAATCTTCTAAGCCATTAAGATTTGTTTTATTAGGTGGACAACCTATTAATGAACCTGTGGTTCAAGCTGCTCCTTTTGTGATGAACTCACAAGCTGAAATTGATCAAACTTTTGAAGATTATCAGTATTGCAAGAATGGTTTTGAGAATGCTAGATATTGGAGATCAGGGCACTGA |
| NbCYSP6 | KX375796.1 | https://www.ncbi.nlm.nih.gov | ATGGCAAATCATAGCTCCACTCTCACCATATCCCTACTTCTCCTCCTCTTCTTCTTCTCCACCTTATCTTCCGCTTCCGACATGTCCATCTTAACCTACGACGAAAACCAACACTTTCGAACAGACGCTGAAGTCATGTCCTTGTACGAGTCATGGCTAGTCGAACATGGAAAATCCTACAACGCCTTAGACGAAAAAGACAAGCGGTTTCAGATCTTTAAAGATAACCTAAGATACATAGATGAACAAAACTCTGTTCCAAACAAGAGTTACAAGCTTGGTTTAACAAAATTTGCTGATCTGACTAACGACGAGTACAGGTCCATGTACTTAGGTACTAAGACCACTGATCGTCGCAGGTTGTTGAAAAACAAAAGCGATCGGTATCTTCCTAAAGTTGGGGATAGCTTGCCTGACTCAGTTGACTGGAGAGAGAAAGGTGTTCTTGTTGGAGTTAAGGATCAAGGAAGCTGTGGGAGTTGTTGGGCATTCTCTGCAATTGCTTCCGTTGAAGCAGTGAACTCGATAGTCACTGGAGATGTGATTTCACTATCGGAGCAAGAGCTGGTTGATTGTGATACTTCCTACAACGACGGTTGCAATGGCGGTCTTATGGACTATGCCTTTGATTTCATCATTAAAAATGGAGGAATTGACACTGAGGAAGACTACCCTTACACAGGCCGTGATGGTAGATGTGACCAGTCAAGGAAAAATGCCAAGGTTGTTACCATAGATGGGTATGAAGATGTTCCTGCAAATAATGAGAAGGCACTGCAAAAGGCTGTTGCAAGTCAACCTGTGAGCATTGCCATTGAAGCTGGTGGCAACGACTTTCAACACTATGTATCGGGTATCTTTACTGGTAAATGTGGGACTGCAGTGGACCATGGTGTGGTAGCTGTTGGATATGGTAGTGAGAATGGCATGGATTATTGGATTATTAGAAACTCTTGGGGTGCTCGTTGGGGAGAACAAGGTTACCTTCGGGTTCAACGTAACGTTGCCAGTTCTAAAGGTTTGTGTGGATTAGCCATAGAGCCTTCTTACCCAGTCAAGACAGGTGTAAATCCCCCTAAACCTGGACCTTCTCCTCCATCTCCAATCAAGCCACCTACTCAGTGTGATGATTATGCTCAATGCCCCGAGGGTACCACTTGCTGTTGTGTCTTTGAATACTATAACTCCTGCTTCTCTTGGGGATGTTGCCCTCTTGAAGGAGCCACTTGCTGTGAAGATCACTACAGTTGTTGCCCACACGACTATCCTGTCTGCAATATTCGTGCAGGCACCTGCTCAATAAGCAAGGACAACCCCCTGGGGGTGAAGGCAATGAAGCACATCCTTGCTGAACCAATTGGGGCCTTCATAAATGGAGGACGGAAGAGCAGTTCTTGA |
| CRP | NC_002356.1 | https://www.ncbi.nlm.nih.gov | ATGACTACTGGTACTCATTCTTGTGAGAAGTGTGCTAACGGTTTCTCTAATGTTATCTGCGTTAGTAAGTATCGCACTAGCGTGTATAAATCTCTGGGTTTGGTTCCTGTTAAATGTCGTCTGCCTGCAGATTGTGGTGTTAGTTGTGGTATGCCTGCGGCGTTTGTTCTGGTGAAAGGACATCCAGAGTTGTCCATGGATGGTTTCTGCGGTGAAAAACATAGAGGTTATGTGGTTTCAGGGGCATGGCGTAGTGCTCAACTTCGCACTTTAAATGCTGAGTTGGATAAGTTGGTTGCCAGGGAGGAATCGCTGCGCTTGCAGATTCGCGGTCTGAATGAGGCCATTAAGACCTCAACCGCACCCGTTTACGCTCCAATTAAACTTCAGAAGCTGAAAGTCGAAGCCTTCAATGTCGACGAAAAAATACAGACTCGCAGCACTGATCTCTGTGATGTGATGACTTCTGTTATGGCTAAGCTGTCAACTGATTCCTCACCGAAGAAGACTCGTGTGGAGTAA |
