# Supplementary material for: NbPIRIN promotes the protease activity of papain-like cysteine protease NbRD21 to inhibit Chinese wheat mosaic virus infection
Source: PLoS Pathog. 2025 Apr 2;21(4):e1013037. doi: 10.1371/journal.ppat.1013037 (PMC11978040; doi:10.1371/journal.ppat.1013037)
Supplement: S2 Table — (DOCX) [file ppat.1013037.s002.docx]

| Number | description | Accession | clone |
| --- | --- | --- | --- |
| 1 | Nicotiana benthamiana papain-like cysteine proteinase 6 mRNA , complete cds | KX375796.1 | 3 |
| 2 | Nicotiana tabacum uncharacterized LOC107789029 (LOC107789029), transcript variant X2,mRNA | [XM_019387911.1](https://www.ncbi.nlm.nih.gov/nucleotide/XM_019387911.1?report=genbank&log$=nucltop&blast_rank=1&RID=18ZVJFXG01R) | 2 |
| 3 | Nicotiana attenuata glutamine synthetase, chloroplastic (LOC109223546), mRNA | [XM_016610800.1](https://www.ncbi.nlm.nih.gov/nucleotide/XM_016610800.1?report=genbank&log$=nucltop&blast_rank=1&RID=1BM2RKKM014) | 1 |
| 4 | Nicotiana tomentosiformis endochitinase A (LOC104109655), mRNA | [XM_009619007.2](https://www.ncbi.nlm.nih.gov/nucleotide/XM_009619007.2?report=genbank&log$=nucltop&blast_rank=1&RID=1CZY1PHG014) | 1 |
| 5 | Nicotiana sylvestris uncharacterized LOC104225600 (LOC104225600), mRNA | [XM_009777441.1](https://www.ncbi.nlm.nih.gov/nucleotide/XM_009777441.1?report=genbank&log$=nucltop&blast_rank=1&RID=1D0ZZ0XB016) | 1 |
| 6 | Nicotiana sylvestris endoglucanase 6-like (LOC104215840), mRNA | [XM_009765761.1](https://www.ncbi.nlm.nih.gov/nucleotide/XM_009765761.1?report=genbank&log$=nucltop&blast_rank=1&RID=1D17893M016) | 1 |
| 7 | Nicotiana tabacum annexin D1-like (LOC107828666), mRNA | [NM_001326207.1](https://www.ncbi.nlm.nih.gov/nucleotide/NM_001326207.1?report=genbank&log$=nucltop&blast_rank=1&RID=1D1KDG1N014) | 1 |
| 8 | Nicotiana tabacum aspartate aminotransferase, cytoplasmic-like (LOC107778680), mRNA | [XM_016598965.1](https://www.ncbi.nlm.nih.gov/nucleotide/XM_016598965.1?report=genbank&log$=nucltop&blast_rank=1&RID=1D1R2075014) | 1 |
| 9 | Nicotiana sylvestris cytochrome b6-f complex iron-sulfur subunit 2, chloroplastic (LOC104226732), mRNA | [XM_009778785.1](https://www.ncbi.nlm.nih.gov/nucleotide/XM_009778785.1?report=genbank&log$=nucltop&blast_rank=1&RID=1D1X23PT016) | 1 |
| 10 | Nicotiana attenuata probable BOI-related E3 ubiquitin-protein ligase 3 (LOC109210670), mRNA | [XM_019374124.1](https://www.ncbi.nlm.nih.gov/nucleotide/XM_019374124.1?report=genbank&log$=nucltop&blast_rank=1&RID=1D22WZFH016) | 1 |
| 11 | Nicotiana attenuata aconitate hydratase, cytoplasmic (LOC109229331), mRNA | [XM_019394735.1](https://www.ncbi.nlm.nih.gov/nucleotide/XM_019394735.1?report=genbank&log$=nucltop&blast_rank=1&RID=1D27BV7D014) | 1 |
| 12 | Nicotiana sylvestris ubiquitin receptor RAD23c-like (LOC104238574), transcript variant X1, mRNA | [XM_009792972.1](https://www.ncbi.nlm.nih.gov/nucleotide/XM_009792972.1?report=genbank&log$=nucltop&blast_rank=1&RID=1D2E3571016) | 1 |
| 13 | Nicotiana benthamiana NbGAPDH-A mRNA for glyceraldehyde 3-phosphate dehydrogenase-A, complete cds | [AB937979.1](https://www.ncbi.nlm.nih.gov/nucleotide/AB937979.1?report=genbank&log$=nucltop&blast_rank=1&RID=1D2HYDYM014) | 1 |
| 14 | Nicotiana tomentosiformis chlorophyll a-b binding protein 40, chloroplastic (LOC104119056), mRNA | [XM_009630460.2](https://www.ncbi.nlm.nih.gov/nucleotide/XM_009630460.2?report=genbank&log$=nucltop&blast_rank=1&RID=1D2REGWR016) | 1 |
| 15 | Nicotiana sylvestris cytochrome b6-f complex iron-sulfur subunit 2, chloroplastic (LOC104226732), mRNA | [XM_009778785.1](https://www.ncbi.nlm.nih.gov/nucleotide/XM_009778785.1?report=genbank&log$=nucltop&blast_rank=1&RID=1D2Y7ACA014) | 1 |
| 16 | Nicotiana sylvestris oxygen-evolving enhancer protein 1, chloroplastic (LOC104219516), mRNA | [XM_009770209.1](https://www.ncbi.nlm.nih.gov/nucleotide/XM_009770209.1?report=genbank&log$=nucltop&blast_rank=1&RID=1D30GRY7016) | 1 |
| 17 | Nicotiana tabacum transcription factor VOZ1-like (LOC107810741), transcript variant X1, mRNA | [XM_016635552.1](https://www.ncbi.nlm.nih.gov/nucleotide/XM_016635552.1?report=genbank&log$=nucltop&blast_rank=1&RID=1D3BKDBF016) | 1 |
| 18 | Nicotiana attenuata gamma carbonic anhydrase 1, mitochondrial-like (LOC109208710), mRNA | [XM_019371851.1](https://www.ncbi.nlm.nih.gov/nucleotide/XM_019371851.1?report=genbank&log$=nucltop&blast_rank=1&RID=1D3N8YUB014) | 1 |
| 19 | Nicotiana sylvestris pyridoxal kinase (LOC104248284), mRNA | [XM_009804516.1](https://www.ncbi.nlm.nih.gov/nucleotide/XM_009804516.1?report=genbank&log$=nucltop&blast_rank=1&RID=FT21RNMC016) | 1 |
| 20 | Nicotiana attenuata rac-like GTP-binding protein 5 (LOC109220368), transcript variant X3, mRNA | [XM_019384832.1](https://www.ncbi.nlm.nih.gov/nucleotide/XM_019384832.1?report=genbank&log$=nucltop&blast_rank=1&RID=FSUR6DYD016) | 1 |
| 21 | Nicotiana benthamiana NbGAPDH-A mRNA for glyceraldehyde 3-phosphate dehydrogenase-A, complete cds | [AB937979.1](https://www.ncbi.nlm.nih.gov/nucleotide/AB937979.1?report=genbank&log$=nucltop&blast_rank=1&RID=FSVUBSPS016) | 1 |
| 22 | Nicotiana benthamiana cytosolic glyceraldehyde-3-phosphate dehydrogenase 1 (GAPC1) mRNA, complete cds | [KM986323.1](https://www.ncbi.nlm.nih.gov/nucleotide/KM986323.1?report=genbank&log$=nucltop&blast_rank=1&RID=FSX2G2XY014) | 1 |

Table S1. The total of 25 positive clones obtained from the Y2H screening with NbPIRIN as bait
