# Supplementary material for: NbPIRIN promotes the protease activity of papain-like cysteine protease NbRD21 to inhibit Chinese wheat mosaic virus infection
Source: PLoS Pathog. 2025 Apr 2;21(4):e1013037. doi: 10.1371/journal.ppat.1013037 (PMC11978040; doi:10.1371/journal.ppat.1013037)
Supplement: S3 Table — (DOCX) [file ppat.1013037.s003.docx]

| Table S2. Detail information of primers | | |
| --- | --- | --- |
| primer name | sequence | Plasmid name |
| S1F | ATGGCCGTGAAATCTGGTTAT | CWMV-CP |
| S1R | ACACTCGAACCTTCCCACTTAAG |  |
| S2F | ACTTACATGCTAGAGGGAGCT | NbPIRIN-QRT |
| S2R | TGGTATGTCCTCATTCAGCAGT | |
| S3F | GTACCAGATTACGCTCATATGATGAGAGCTATTTTTAAAC | AD-PIRIN |
| S3R | ATGCCCACCCGGGTGGAATTCTCAGTGCCCTGATCTCCAAT |  |
| S4F | GGGGACAAGTTTGTACAAAAAAGCAGGCTTCATGAGAGCTATTTTTAAAC | Don207-PIRIN |
| S4R | GGGGACCACTTTGTACAAGAAAGCTGGGTCGTGCCCTGATCTCCAATATC |  |
| S5F | CTGGTTCCGCGTGGATCCATGAGAGCTATTTTTAAAC | PIRIN-GST |
| S5R | GGCCGCTCGAGTCGACCCGGGTCAGTGCCCTGATCTCCAAT |  |
| S6F | TCAGAGGAGGACCTGCATATGATGTCCATCTTAACCTACGA | BD-NbCYSP6 |
| S6R | TCGACGGATCCCCGGGAATTCTCAAGAACTGCTCTTCCGTCCT |  |
| S7F | CTGGTTCCGCGTGGATCCATGGCAAATCATAGCTCCACT | NbCYSP6-GST |
| S7R | AAGAATGCGGCCGCTCAAGAACTGCTCTTCCGTCCT |  |
| S8F | GGGGACAAGTTTGTACAAAAAAGCAGGCTTCATGGCAAATCATAGCTCCACT | Don207-NbCYSP6 |
| S8R | GGGGACCACTTTGTACAAGAAAGCTGGGTCAGAACTGCTCTTCCGTCCT |  |
| S9F | TCAGAGGAGGACCTGCATATGATGACTACTGGTACTCATTC | BD-CRP |
| S9R | TCGACGGATCCCCGGGAATTCTTACTCCACACGAGTCTTCTTCGGT | |
| S10F | TAATAAGATATCATGACTACTGGTACTCATTC | CRP-His |
| S10R | TAATAACTCGAGCTCCACACGAGTCTTCTTCGGT | |
| S11F | GGGGACAAGTTTGTACAAAAAAGCAGGCTTCATGACTACTGGTACTCATTC | Don207-CRP |
| S11R | GGGGACCACTTTGTACAAGAAAGCTGGGTCCTCCACACGAGTCTTCTTCGGT | |
| S12F | AGCCCAGATCAACTAGTATGGCAAATCATAGCTCCACT | NbCYSP6-GFP |
| S12R | CTTGCTCACCATGGATCCAGAACTGCTCTTCCGTCCT | |
| S13F | GTACCAGATTACGCTCATATGATGTCTGCCGCCGCCTGCGT | AD-TaPIRIN |
| S13R | ATGCCCACCCGGGTGGAATTCTCACTGGCTAGAGGTCCA |  |
| S14F | CTGGTTCCGCGTGGATCCATGTCTGCCGCCGCCTGCGT | TaPIRIN-GST |
| S14R | GGCCGCTCGAGTCGACCCGGGTCACTGGCTAGAGGTCCAGCC |  |
| S15F | GGGGACAAGTTTGTACAAAAAAGCAGGCTTCATGTCTGCCGCCGCCTGCGT | Don207-TaPIRIN |
| S15R | GGGGACCACTTTGTACAAGAAAGCTGGGTCTCACTGGCTAGAGGTCCA |  |
| S16F | CCTTAATTAAGGTGTGGAGGAGGCGGAGGA | BSMV-TaPIRIN-1 |
| S16R | TTTTCCTTTTGCGGCCGCTGGATGATCGGAAAAGCCA | |
| S17F | CCTTAATTAAGGAACGGCTTCGAGATGG | BSMV-TaPIRIN-2 |
| S17R | TTTTCCTTTTGCGGCCGC ATGTGACTATCATGCAATACT |  |
| S18F | GTACCAGATTACGCTCATATGATGGCGGACATGTCGATCGT | AD-TaCYSP6-NoNSP |
| S18R | ATGCCCACCCGGGTGGAATTCTTACGCGCTGCTCTTCTTGC | |
| S19F | TCAGAGGAGGACCTGCATATGATGGCGGACATGTCGATCGT | BD-TaCYSP6-NoNSP |
| S19R | TCGACGGATCCCCGGGAATTCTTACGCGCTGCTCTTCTTGC |  |
| S20F | GGGGACAAGTTTGTACAAAAAAGCAGGCTTCATGAGGAGCTCCATGGCTCT | Don207-TaCYSP6 |
| S20R | GGGGACCACTTTGTACAAGAAAGCTGGGTCCGCGCTGCTCTTCTTGC |  |
| S21F | CCTTAATTAAGGATGATCCCTCTGTCCGAGCA | BSMV-TaCYSP6 |
| S21R | TTTTCCTTTTGCGGCCGCCTGGAATGCCCTGCCACCA |  |
| S22F | TGGAGGTACATTTAAGCTGACAC | NbUBC |
| S22R | TCACAGAGCAAAGACTGGATTG |  |
