# Supplementary figures and images for: NbPIRIN promotes the protease activity of papain-like cysteine protease NbRD21 to inhibit Chinese wheat mosaic virus infection
Source: PLoS Pathog. 2025 Apr 2;21(4):e1013037. doi: 10.1371/journal.ppat.1013037 (PMC11978040; doi:10.1371/journal.ppat.1013037)

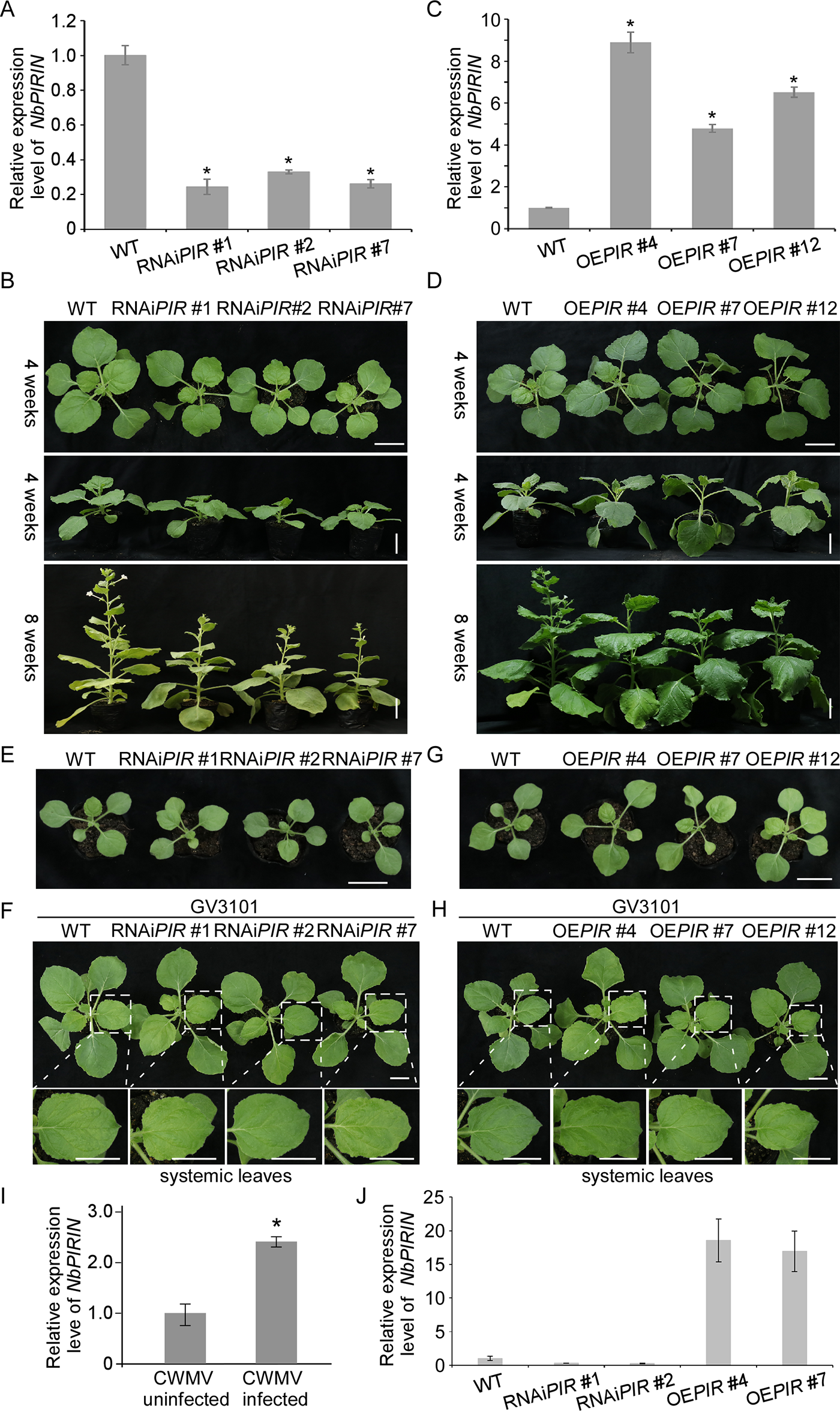

Supplement: S1 Fig — An asterisk above the bar indicates a significant difference between the two treatments (*, P ≤ 0.05 by Student’s t-test). (B) Vegetative growth of wild type and three NbPIRIN silencing lines at 4 weeks and 8 weeks. (C) Quantitative RT-PCR analysis of NbPIRIN relative expression level in wild type and three NbPIRIN-overexpressing transgenic lines. An asterisk above the bar indicates a significant difference between the two treatments (*, P ≤ 0.05 by Student’s t-test). (D) Vegetative growth of wild type and three NbPIRIN-overexpressing transgenic lines at 4 weeks and 8 weeks. (E) Photograph of the whole plants of WT, RNAiPIR#1, RNAiPIR#2 and RNAiPIR#7 at the age of inoculation. (F) Photograph of WT, RNAiPIR#1, RNAiPIR#2 and RNAiPIR#7 plant leaves after 21 days post inoculation with agroinfiltration GV3101. (G) Photograph of the whole plants of WT, OEPIR#4, OEPIR#7 and OEPIR#12 at the age of inoculation. (H) Photograph of WT, OEPIR#4, OEPIR#7 and OEPIR#12 plant leaves after 21 days post inoculation with agroinfiltration GV3101. (I) Quantitative RT-PCR analysis of NbPIRIN relative expression level in the CWMV-infected and uninfected wheat leaves. An asterisk above the bar indicates a significant difference between the two treatments (*, P ≤ 0.05 by Student’s t-test). (J) Quantitative RT-PCR analysis of NbPIRIN relative expression level in wild type, three NbPIRIN silencing lines and three NbPIRIN-overexpressing transgenic lines. An asterisk above the bar indicates a significant difference between the two treatments (*, P ≤ 0.05 by Student’s t-test). (TIF) [file ppat.1013037.s004.tif]

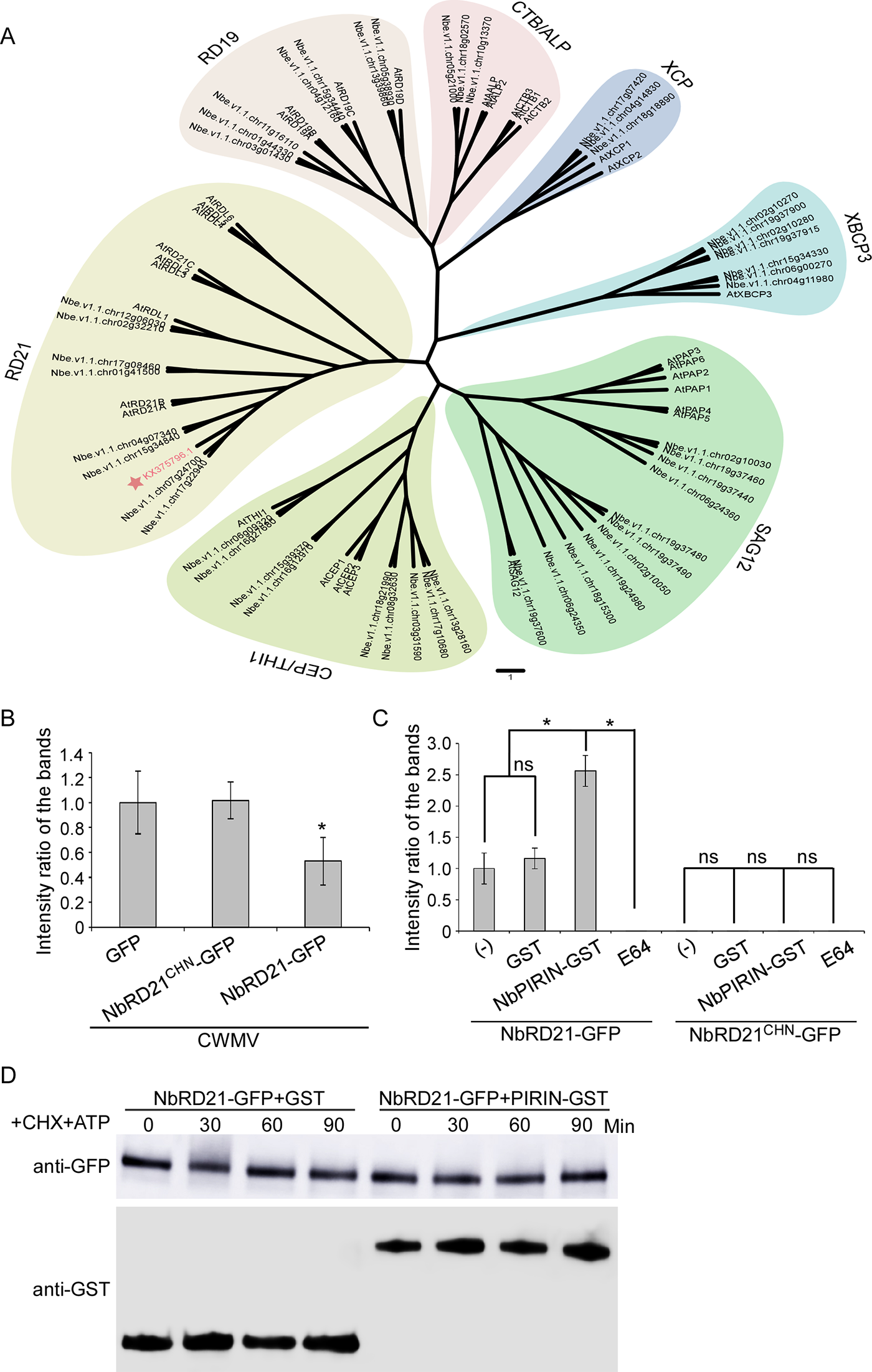

Supplement: S2 Fig — Each group is represented by a different colour. Stars represent NbRD21. First, the NbRD21 protein sequence is retrieved in the phylogenetic genome using Blastp with evalue =1E-7. Phylogenetic analysis is performed with phylosuite software after integration of Arabidopsis data, and finally visualization is performed with Figtree software. (B) Bar plot of the intensity ratio of the bands in Fig 2F calculated by Image J software. There were three biological replicates of each treatment. An asterisk above the bar indicates a significant difference between the two treatments (*, P ≤ 0.05 by Student’s t-test). (C) Bar plot of the intensity ratio of the bands in Fig 2G calculated by Image J software. Experiment in Fig 2G was repeated three times, and the intensity ratio was obtained according to three repeated calculations. An asterisk above the bar indicates a significant difference between the two treatments (*, P ≤ 0.05 by Student’s t-test). (D) Western blot analysis of NbRD21-GFP using a GFP specific antibody at different times after the addition of the protein translation inhibitor cycloheximide (CHX) and adenosine triphosphate (ATP), Protein load of GST and PIRIN-GST were analyzed by Western blotting with anti-GST antibodies (HT601, TransGen Biotech, China). (TIF) [file ppat.1013037.s005.tif]

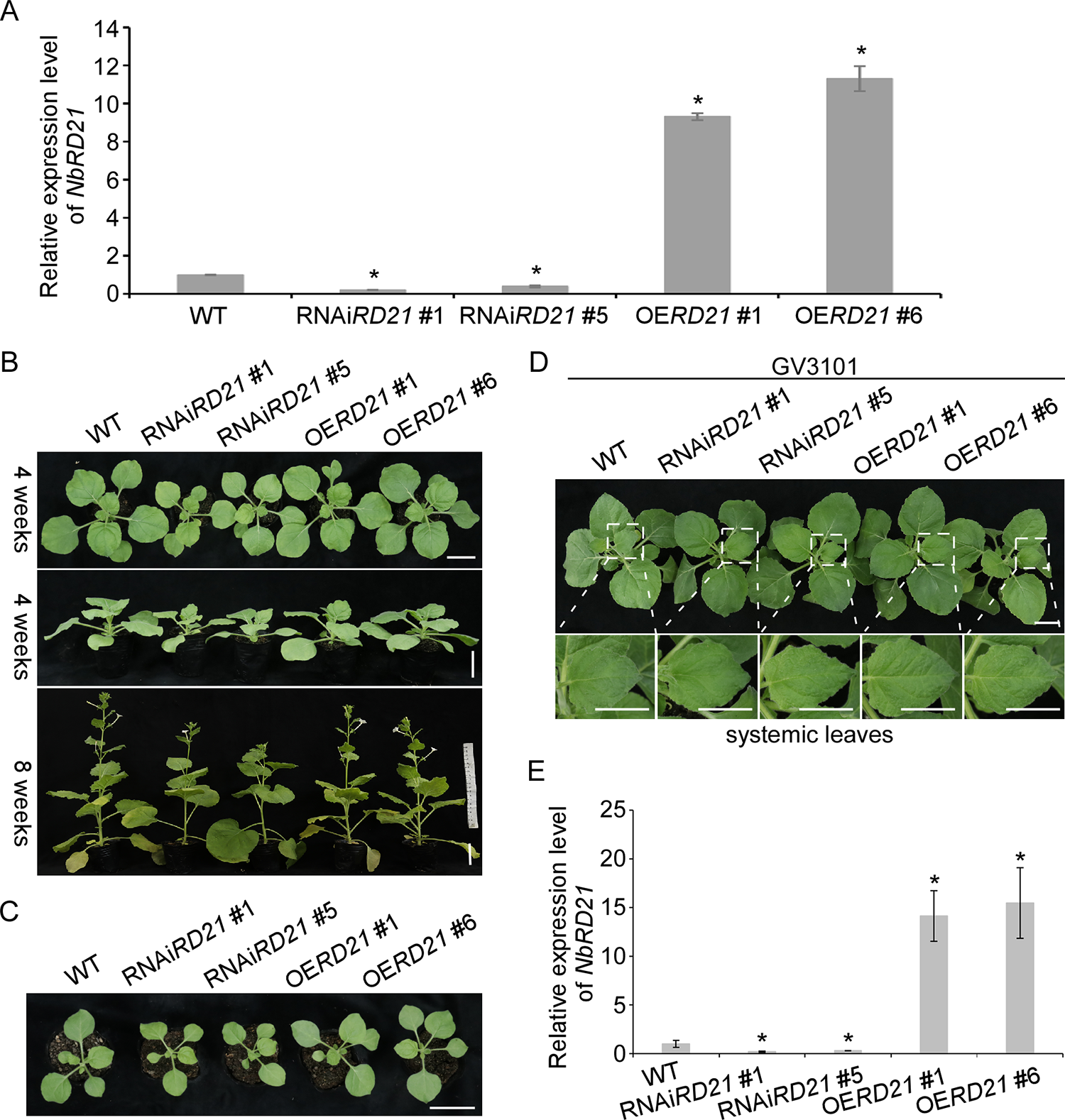

Supplement: S3 Fig — An asterisk above the bar indicates a significant difference between the two treatments (*, P ≤ 0.05 by Student’s t-test). (B) Vegetative growth of wild type, NbRD21 silencing lines and NbRD21-overexpressing transgenic lines at 4 weeks and 8 weeks. (C) Photograph of the whole plants of WT, RNAiRD21#1, RNAiRD21#5, OERD21#1 and OERD21#6 at the age of inoculation. (D) Photograph of WT, RNAiRD21#1, RNAiRD21#5, OERD21#1 and OERD21#6 plant leaves after 21 days post inoculation with agroinfiltration GV3101. (E) Quantitative RT-PCR analysis of NbRD21 relative expression level in WT, RNAiRD21#1, RNAiRD21#5, OERD21#1 and OERD21#6. An asterisk above the bar indicates a significant difference between the two treatments (*, P ≤ 0.05 by Student’s t-test). (TIF) [file ppat.1013037.s006.tif]

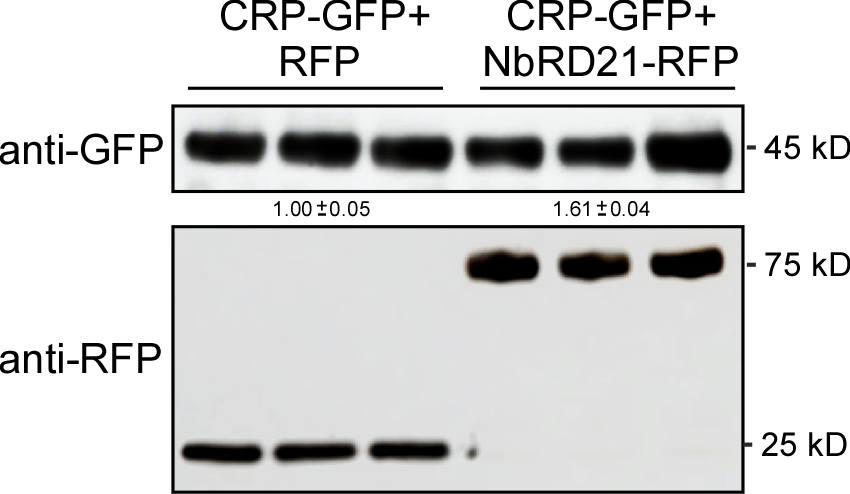

Supplement: S4 Fig — Protein load of RFP and NbRD21-RFP were analyzed by Western blotting with anti-RFP antibodies (6g6, Chromoteck, USA). (TIF) [file ppat.1013037.s007.tif]

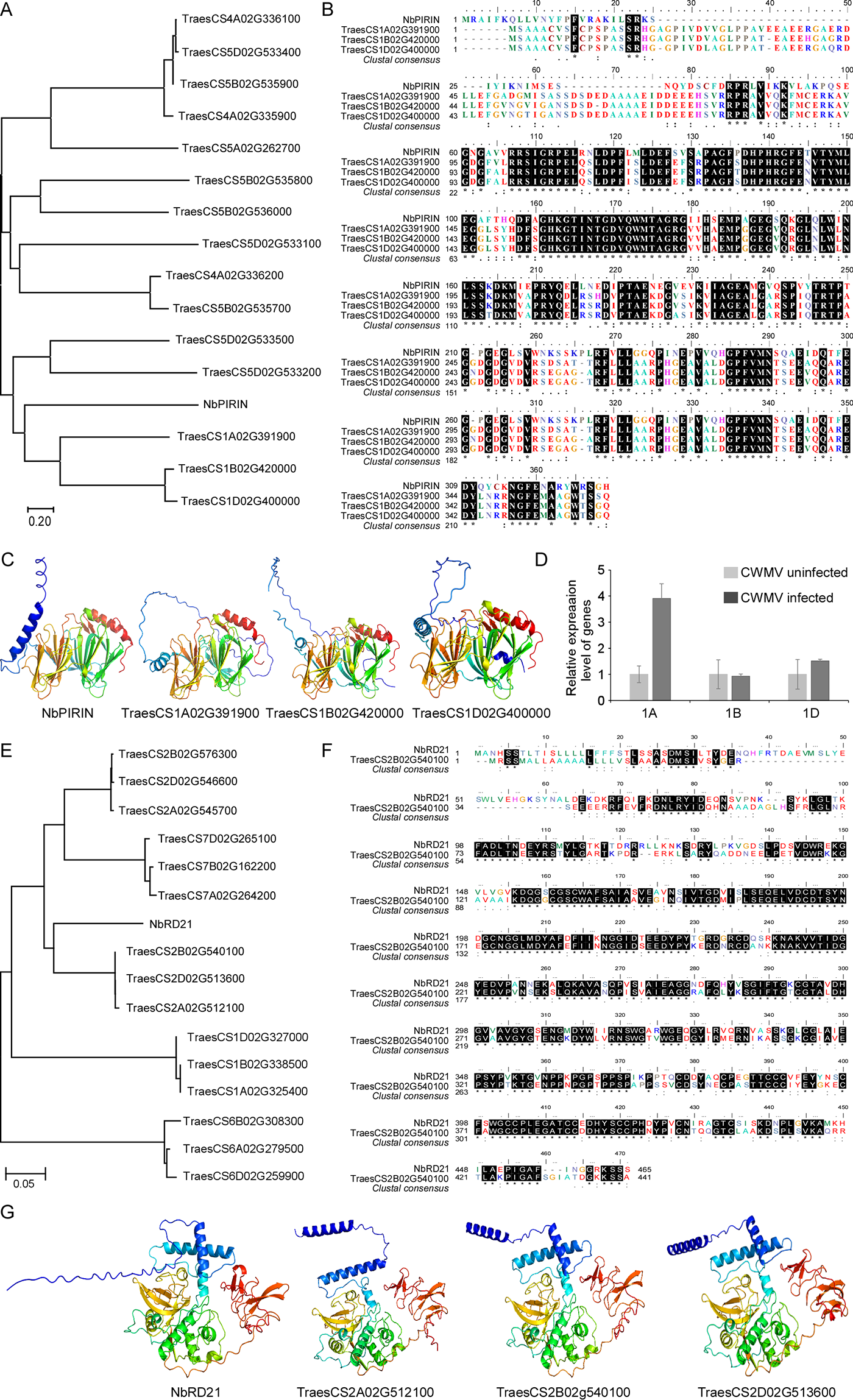

Supplement: S5 Fig — (B) Full length PIRIN amino acid sequences from T. aestivum and N. benthamiana were aligned using Bioedit software. The overlined areas represent the PIRIN domain, and the numbers represent homology. (C) Structural homology searches made between NbPIRIN and three TaPIRIN proteins showed that three TaPIRIN have similar protein structure to NbPIRIN. (D) Quantitative RT-PCR analysis of TraesCS1A02G391900, TraesCS1B02G420000 and TraesCS1D02G40000 in the CWMV-infected and uninfected wheat leaves. An asterisk above the bar indicates a significant difference between the two treatments (*, P ≤ 0.05 by Student’s t-test). (E) Phylogenetic analysis of the RD21 proteins from T. aestivum and N. benthamiana constructed by the neighbor-joining method in MEGA-7. (F) Full length RD21 amino acid sequences from T. aestivum and N. benthamiana were aligned using Bioedit software. The overlined areas represent the Papain family cysteine protease domain, and the numbers represent homology. (G) Structural homology searches made between NbRD21 and three TaRD21 proteins showed that three TaRD21 have similar protein structure to NbRD21. (TIF) [file ppat.1013037.s008.tif]

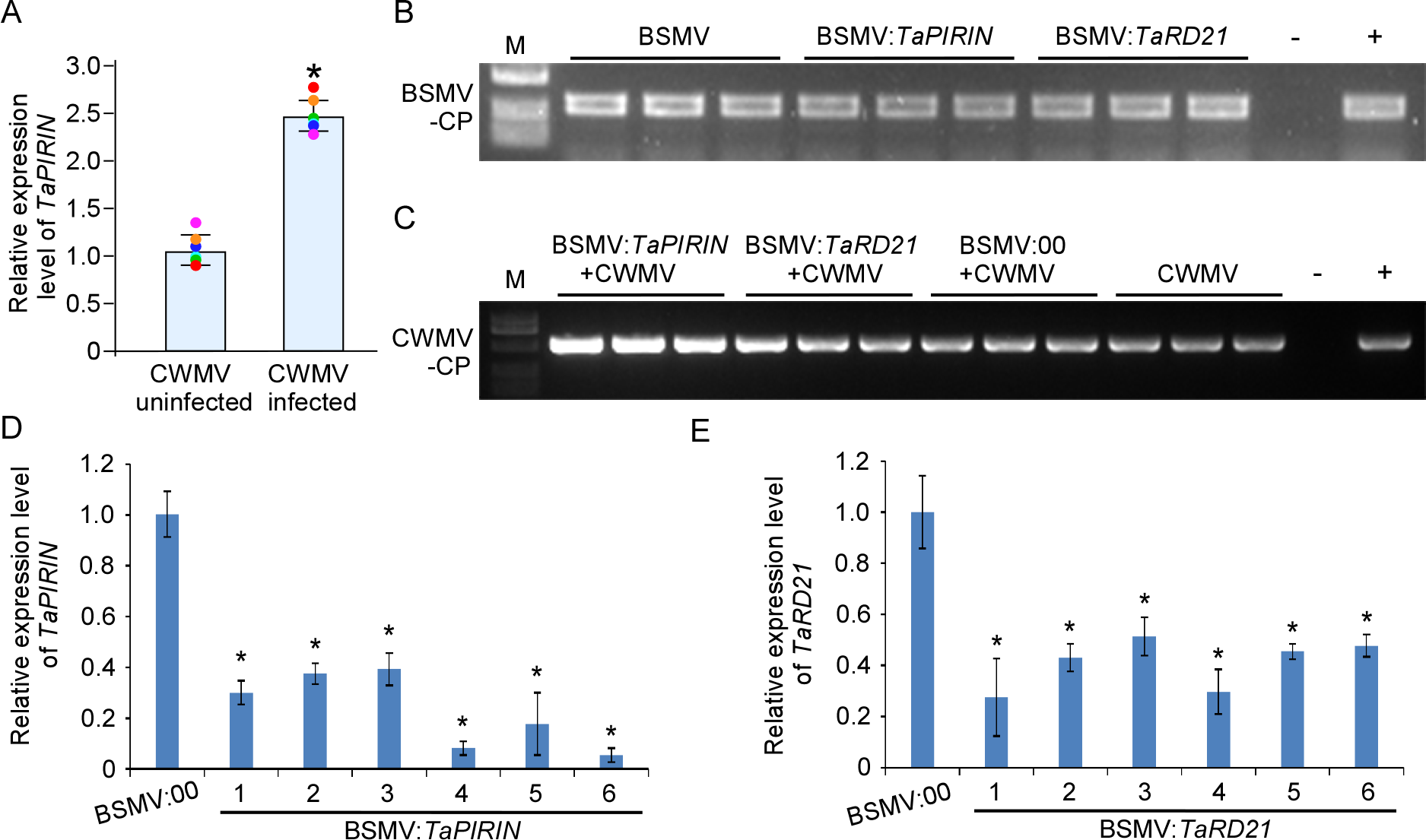

Supplement: S6 Fig — (A) Quantitative RT-PCR analysis of TaPIRIN relative expression level in the CWMV-infected and uninfected wheat leaves. An asterisk above the bar indicates a significant difference between the two treatments (*, P ≤ 0.05 by Student’s t-test). (B) Confirmation of BSMV infection in the BSMV-inoculated, BSMV:TaPIRIN -inoculated or BSMV:TaRD21 -inoculated wheat plants through RT-PCR at 7 dpi. (C) Confirmation of CWMV infection in the CWMV-inoculated BSMV:00 and CWMV-inoculated, BSMV:TaPIRIN and CWMV-inoculated, or BSMV:TaRD21 and CWMV-inoculated wheat plants through RT-PCR at 7 dpi. (D) Confirmation of TaPIRIN silencing in the BSMV:00 and BSMV:TaPIRIN CWMV-inoculated wheat plants through RT-PCR at 7 dpi. (E) Confirmation of TaRD21 silencing in the BSMV:00 and BSMV:TaRD21 CWMV-inoculated wheat plants through RT-PCR at 7 dpi. (TIF) [file ppat.1013037.s009.tif]
